# Deletion of *pagL* and *arnT* genes involved in LPS structure and charge modulation in the *Salmonella* genome confer reduced endotoxicity and retained efficient protection against wild-type *Salmonella* Gallinarum challenge in chicken

**DOI:** 10.1186/s13567-024-01413-8

**Published:** 2025-01-04

**Authors:** Ram Prasad Aganja, Jun Kwon, Amal Senevirathne, John Hwa Lee

**Affiliations:** 1 College of Veterinary Medicine, Jeonbuk National University, Iksan Campus, Iksan, 54596 Republic of Korea; 2 College of Veterinary Medicine and Institute of Animal Transplantation, Jeonbuk National University Campus, Iksan, 54596 Republic of Korea

**Keywords:** Fowl typhoid, *Salmonella* Gallinarum, genetic modification, lipopolysaccharide biosynthesis, vaccine

## Abstract

Fowl typhoid (FT) poses a significant threat to the poultry industry and can cause substantial economic losses, especially in developing regions. Caused by *Salmonella* Gallinarum (SG), vaccination can prevent FT. However, existing vaccines, like the SG9R strain, have limitations, including residual virulence and potential reversion of pathogenicity. This study aims to develop safer and more effective SG vaccine strains through targeted genetic modifications, focusing on genes involved in lipopolysaccharide (LPS) biosynthesis and modification. We evaluated two novel mutant SG strains, JOL3015 and JOL3016, carrying in-frame deletions in Δ*lon*Δ*rfaL*Δ*arnT* and Δ*lon*Δ*rfaL*Δ*pagL*, respectively. Intramuscular immunisation of 4-week-old young birds with JOL3015 and JOL3016 strains showed minimal impact on their growth. However, the immunisation significantly increased antigen-specific IgY, sIgA secretion, and CD4^+^ and CD8^+^ T-cell responses while inducing lower pro-inflammatory cytokine levels than SG9R. Histopathological evaluations revealed substantial protection in the immunised birds, with minimal tissue damage and inflammatory responses, thus reducing the in vivo bacterial burden. Furthermore, none of the immunised birds died. This outcome highlights the significant safety and protection the selected genetic modifications conferred. Our results indicate that JOL3016 provided comparable protective outcomes on par with SG9R, yet with significantly lower endotoxicity responses during the lethal challenge with SG WT JOL422. The novel detoxified SG strains, particularly JOL3016, offer a promising alternative to existing vaccines for FT. They provide effective protection with minimal impact on poultry growth, thereby minimising the risks associated with reversion and endotoxicity. The study highlights the potential of genetically engineered vaccine strains in improving poultry health and productivity, emphasising the importance of continued research.

**Supplementary Information:**

The online version contains supplementary material available at 10.1186/s13567-024-01413-8.

## Introduction

Fowl typhoid (FT), caused by *Salmonella enterica* serovar Gallinarum (*Salmonella* Gallinarum, SG), is a severe systemic disease affecting chickens of all age groups. According to some studies, SG has a global prevalence of 8.54%, with Asia ranking at the top [1]. Various factors, including host age, host susceptibility, nutrition, flock management, and bacterial virulence, influence the severity of the disease. The disease imposes a significant threat to the poultry industry, causing up to 100% mortality and substantial economic losses [2]. Current control measures include strict biosecurity regulations, antibiotic use, and vaccination. However, maintaining biosecurity is costly and challenging for poultry operations, while long-term antibiotic use can lead to the development of multi-drug resistant strains. Consequently, vaccination is one of the most effective control strategies, with options including live, inactivated, and subunit vaccines. Although FT has been eradicated from commercial poultry in developed countries, it remains widespread in most developing countries. This ongoing issue underscores the need for effective and accessible vaccination strategies to mitigate the impact of FT on global poultry production [3].

The live attenuated SG9R strain, a semi-rough strain with limited information on its attenuation, serves as a commercial vaccine for FT. However, SG9R has been reported to cause systemic disease, liver and spleen pathology, and bacterial persistence for several weeks in young chickens, which could inevitably affect the productivity of young birds [4]. Furthermore, SG9R vaccination has been associated with residual virulence in newly hatched chickens, limited protection, and vertical transmission [5]. The disease remains prevalent among poultry flocks despite Korea’s early adoption of a control and eradication policy for FT in the 1970s [6].

This study proposes that a safer SG vaccine can be developed through bacterial strain manipulation using genetic engineering to address this gap. It is well-established that bacterial lipopolysaccharides (LPS) initiate pro-inflammatory immune responses and endotoxicity, which can be lethal to the host, especially at a young age [7]. Lipid A and its acyl chains in LPS play a central role in triggering inflammatory cytokines. Hexa-acylated lipid A stimulates a maximum pro-inflammatory response via the TLR4-MD2-CD14 pathway, while tetra- or penta-acylated species significantly reduce immunostimulatory responses [8]. Thus, lipid A-derived endotoxicity can be mitigated by its structural remodelling. In the present context, the PhoP/PhoQ-activated gene (*pagL*) encodes deacylase. This encoding modifies lipid A by removing R-3-hydroxy myristate attached at position 3, thus maintaining bacterial virulence. Hence, *pagL* deletion can confer detoxification of lipid A, reducing endotoxicity [9].

Similarly, *arnT* (l-Ara4N transferase) modifies LPS by adding 4-amino-4-deoxy-l-arabinose (l-Ara4N) to lipid A’s phosphate groups, altering the charge and structure of the LPS. This modification contributes to bacterial survival and immune evasion [10]. Hence, *arnT* represents a potential target for regulating the virulence of SG strains. Furthermore, by precisely and permanently deleting such genes, there is no risk of SG wild-type (WT) strains reverting to a virulent form, making this strategy safe and effective for developing vaccine candidates. Therefore, remodelling the LPS structure holds promising potential for generating avirulent SG strains for vaccine development.

Furthermore, the serological diagnosis of *Salmonella* infection relies on detecting LPS-specific antibodies against the O-antigen. However, this method can often be hindered by field infections, making it challenging to differentiate between infected and vaccinated animals (DIVA). The DIVA concept is crucial for implementing effective vaccination strategies. Monitoring salmonellosis and ensuring ideal vaccination necessitates the capability to differentiate infected from vaccinated animals, a feat that can be achieved through LPS truncation via O-antigen modification. Thus, targeting the deletion of *rfaL*, which encodes O-antigen ligase, aims to lower LPS-specific antibodies compared to wild-type infection [11]. Ultimately, this strategy aids in differentiating animals that are infected from those that have been vaccinated by quantifying antibody levels using enzyme-linked immunosorbent assay (ELISA). The Lon protease serves as a global regulator that controls the expression of virulence genes located in *Salmonella* pathogenicity island I (SPI-1) during the early stages of systemic infection. The dysregulation of Lon protease, a negative regulator of SPI-1 genes, causes an increase in the expression and coordination of early virulence genes [12]. However, attenuating SG through *lon* gene deletion renders the strain hyper-immunogenic with reduced virulence [13]. This targeted genetic modification not only enhances the immunogenicity of the strain but also contributes to its safety profile, making it a promising candidate for vaccine development against *Salmonella* infection.

The study’s objective was to comprehensively evaluate the safety and protective efficacy of attenuated SG strains engineered through targeted deletion of the *lon* gene to reduce virulence. Additionally, *rfaL* gene deletion was undertaken to enhance the capability of monitoring salmonellosis using the DIVA principle. The strains underwent detoxification processes to yield SG strains with Δ*lon*Δ*rfaL*Δ*pagL* and Δ*lon*Δ*rfaL*Δ*arnT* modifications. The study conducted comparative assessments to examine the protective potential of these engineered strains against wild-type challenge. The results showed a significant improvement in safety and efficacy compared to SG9R, a commercial vaccine strain. These findings underscore the potential of genetically engineered SG strains as viable candidates for advanced vaccine development, offering enhanced safety, efficacy, and monitoring capabilities in combating *Salmonella* infection in poultry populations.

## Materials and methods

### Bacterial strains, plasmids, and growth conditions

All bacterial strains were routinely grown in Luria Bertani (LB) medium (Becton Dickinson, Sparks, MD, USA) with agitation at 37 °C using appropriate antibiotics where applicable. The strains and plasmids used are listed in Table 1. The SG 914 strain (*Δlon*) [14] was engineered to develop the SG JOL3015 and SG JOL3016 strain by deleting *ΔrfaL ΔarnT* and *ΔrfaL* Δ*pagL*, respectively, using the λ red recombination technique [15]. Briefly, the parent SG strain was transformed with a helper plasmid, pKD46, and induced to express recombinase with L-arabinose for homologous recombination. The linear DNA cassette of the *catR* gene flanked by a *rfaL* gene homologous sequence was amplified from pKD3 and electroporated (Harvard Apparatus, USA) in pKD46-transformed *Salmonella*. The *rfaL*-deleted mutant colonies were screened by plating on LB media containing chloramphenicol. Colonies were confirmed by inner primers and transformed with pCP20 plasmid to eliminate the FRT-flanked *catR* through flippase production. The deletion of *catR* was confirmed using flanking primers (listed in Table 2). The same procedure was repeated to include the deletion of *arnT* and *pagL* in their respective strains. A commercially available vaccine SG9R was procured (9R VAC, Komipharm International Co. Ltd., Siheung, Korea) for the comparative study.

**Table 1:** **List of bacterial strains and plasmids**

Bacteria/plasmid	Genotypic characteristics	References
*S*. Gallinarum		
JOL422	Wild type	Lab stock
JOL914	*Δlon*	Lab strain
JOL3015	*Δlon ΔrfaL ΔarnT*	This study
JOL3016	*Δlon ΔrfaL ΔpagL*	This study
pKD46	oriR101-repA101ts; encodes λ red genes (*exo*,* bet*,* gam*); native terminator (tL3); arabinose-inducible promoter for expression (ParaB); *bla*	[21]
pKD3	oriR6Kgamma, *bla* (ampR), *rgnB* (Ter), *cat*^*R*^, FRT	[21]
pCP20	Helper plasmid contains a temperature-inducible *flp* gene for removing the FRT flanked chloramphenicol gene	[36]

**Table 2:** **List of primers**

Gene	Primer	5′–3′ sequences	References
Gel deletion			
*lon-pKD3*	Sense	GGTATGGAGCACAGCTATACTATCTGATTACCTGGCGGACACTAAACTAAGTGTAGGCTGGAGCT	This study
	Antisense	CGAAATAGCCTGCCAGCCCTGTTTTTATTAGCGCTATTTGCGCGAGGTCAATGGGAATTAGCCATG	
*rfaL*-pKD3	Sense	TTTGGAAAGATTCATTAAAGAGACTCTGTCTCATCCCAAACCTATTGTGGGTGTAGGCTGGAGCTGCTTC	This study
	Antisense	CCTGATGATGGAAAACGCGCTGATACCGTAATAAGTATCAGCGCGTTTTTATGGGAATTAGCCATGGTCC	
*pagL*-pKD3	Sense	AATTTTAAATATGTTAGCCGGTTAAAAATAACTATTGACATTGAAATGGTGTGTAGGCTGGAGCTGCTTC	This study
	Antisense	CGGTGATTAATTACTCCTTCAGCCAGCAACTCGCTAATTGTTATTCAACTATGGGAATTAGCCATGGTCC	
*arnT*-pKD3	Sense	GAGCTGACCGCCAACGCTGAGCAGACTGGCAAGCACCAGAATGACGCCGAGTGTAGGCTGGAGCTGCTTC	This study
	Antisense	ATCCCTGGCCGTGAAGGTTGGCTGGGGTGCCAACAGGCAGCGAGCGCCTCATGGGAATTAGCCATGGTCC	
*lon-inner*	Sense	AATCTGCACGACTACCTCGG	This study
	Antisense	GATTACCGGTCAGGCAGGAA	
*lon-outer*	Sense	CAGGAGTTCTTACAGGTAGA	This study
	Antisense	CCACACTCCGCTGTAGGTGA	
*rfaL*-inner	Sense	ACAAGTTTAGGACTTCGCTGCC	[15]
	Antisense	CAGAATGGTATTATGCGGACCG	
*rfaL*-outer	Sense	GCA GCG TTT CGA GGA ACA AA	[15]
	Antisense	TCG TAT CGG TTG ATA CCG GC	
*pagL*-inner	Sense	CAGATCTCTTTTGCTGCGGG	[15]
	Antisense	AAAAGCCCCAAAGTTCCAGC	
*pagL*-outer	Sense	TGGATGTGCCTGAACAACACT	[15]
	Antisense	TTAGCCTCCCTGTCGCCATA	
*arnT*-inner	Sense	GCAACGCGGTACGTTTATCC	This study
	Antisense	GAAACGCGCTATGCCGAAAT	
*arnT*-outer	Sense	GAGCTGACCGCCAACGCTGA	This study
	Antisense	GAAACGCGCTATGCCGAAAT	

### Bacterial growth kinetics of the engineered *Salmonella *Gallinarum strains

Growth kinetics were evaluated using SG mutant strains and the commercial SG9R strain. Overnight-grown bacterial cultures were used as 1% v/v inoculum into 50 mL of LB broth. The cultures were incubated at 37 °C in a shaking incubator at 200 rpm. Optical density at 600 nm (OD_600_) was measured every 4 h in a 96-well plate (200 µL) using a spectrophotometer (Tecan, Seestrasse, Switzerland). Samples were withdrawn every 4 h and used in colony counting after preparing serial dilutions. Plates containing 30–300 colonies were counted to determine CFUs.

### Auto-aggregation assay

The clustering ability of bacteria was assessed using an auto-aggregation assay. Overnight bacterial cultures were prepared and inoculated at a 1:100 dilution in LB broth. After incubating the cultures at 37 °C for 24 h, the upper layer was collected without disturbing the cultures. This layer was then used to measure the optical density at OD_600_ nm. Subsequently, the culture was resuspended by vortexing and used to take the second absorbance measurements under the same absorbance conditions. The level of auto-aggregation was determined as a percentage using the formula: [(OD_600_ post-resuspension – OD_600_ pre-resuspension)/ OD_600_ post-resuspension] × 100.

### Hemolysis assay

The wild-type and mutant strains were grown as overnight cultures. Subsequently, cells were harvested through centrifugation at a rate of 8000 rpm for 10 min. Supernatants were filtered through 0.2 μm membrane filters (BioFACT, Parit Jamil, Malaysia). The sterile solutions were mixed with 10% chicken red blood cell (RBC) suspension at a 4:1 ratio and incubated in a shaking incubator at 37 °C for 12 h. A control was prepared by adding LB broth to the RBC suspension at the same ratio. After incubation, the suspensions were centrifuged at 2000 rpm for 5 min [16]. Hemolysis rates were determined by measuring absorbance at 570 nm using a multi-well plate reader (Tecan, Männedorf, Switzerland).

### Acriflavine agglutination test

The acriflavine agglutination test determined the lack of O-antigen components and confirmed the rough phenotype [17]. Bacteria cultures were grown on LB agar plates for 24 h, with selected bacterial colonies collected and mixed into 30 µL of 0.2% acriflavine solution (Sigma, Missouri, USA) on glass slides. Cells were gently mixed, interacted for 2 min, and observed under a microscope at 40 × magnification by the naked eye.

### Western blot of lipopolysaccharides

Following the manufacturer’s guidelines, bacterial lipopolysaccharides were extracted using a phenol-based method via an LPS extraction kit (iNtRON Biotechnology, Seoul, South Korea). The LPS samples were separated on 12% sodium dodecyl sulfate-polyacrylamide gel electrophoresis (SDS-PAGE). Western blotting was performed using a mouse monoclonal antibody against *Salmonella* O-antigen at 1:1000 (cat. no. 10R-S103b, Fitzgerald, MA, USA) and goat anti-mouse IgG-HRP conjugate at 1:5000 dilution (cat. no. 1030-05, SouthernBiotech, Birmingham, AL 35209 USA). All steps were conducted according to a previously described procedure [18].

### Adhesion, invasion, and stress survival

Hela and chicken peripheral blood mononuclear cells (PBMCs) were used in vitro to evaluate the adhesion and invasion capability of SG strains. Overnight cultures of bacterial cells were re-inoculated to LB medium as 1% inoculum and incubated for 3 h to reach 0.4–0.6 absorbance at OD_600_. Cells were collected by centrifugation at 12 000 × *g* for 5 min and washed with phosphate-buffered saline (PBS). Following standard procedure, blood was collected from the bird’s wing vein, and PBMCs were isolated using Ficoll-Paque PLUS density gradient media (Cytiva, Uppsala, Sweden) [19]. PBMCs (2 × 10^6^) and Hela cells (2 × 10^5^) were cultured in a 12-well plate with RPMI medium, 10% heat-inactivated foetal bovine serum (FBS), and 1% penicillin-streptomycin. The cells were then incubated at 37 °C in a humidified 5% CO_2_ incubator.

The PBMCs were incubated for 5 h to facilitate attachment, after which the media was changed to retain adherent PBMCs. The adherent PBMCs and Hela cells were then infected with SG WT, SG9R, and the attenuated strains at 40 multiplicity of infection (MOI), 30 min for adhesion and 2.5 h for invasion. The non-infected bacteria were eliminated by 2 h gentamycin treatment (100 µg/mL). Adhered or invaded cells were retrieved by lysis of monolayers using 0.25% Triton X-100, and the bacterial enumeration was done by counting on Brilliant Green Agar (BGA) plates (BD, Difco). To conduct the stress survival assay, bacterial strains grown at the log phase were subjected to acidic stress at pH 4.0 and oxidative stress at 1 mM and 5 mM H_2_O_2_ for 30 min. The survival rate was evaluated based on the initial inoculum by plating on BGA at 10-fold dilution.

### Cell survival and cytotoxicity assay

*Salmonella*-induced cell cytotoxicity was assessed using an IncuCyte live imaging system (Essen Bioscience, MI, USA). HeLa cells were seeded at 5 × 10⁵ cells/mL in 12-well plates. Cells were infected with *Salmonella* at a MOI of 40 for 2 h and washed twice with PBS. Non-infected bacterial cells were eliminated with gentamycin (100 µg/mL) treatment for 2 h. The cells were then treated with propidium iodide (5 µL/mL, cat. no. 556463, BD Biosciences, California, USA) and monitored via imaging at 6 h intervals over 24 h.

### Safety evaluation of detoxified SG strains in chicken

The safety of detoxified SG strains was assessed in female brown-layer chickens following intramuscular (IM) injection. Four-week-old chickens (*n* = 12) were inoculated with either the SG wild-type (WT JOL422), attenuated strains JOL3015 and JOL3016, or a commercial vaccine strain, SG9R. Birds were monitored for morbidity and mortality associated with FT. Clinical parameters such as body temperature, abnormal behaviour, anorexia, and feed intake were observed to detect any adverse effects caused by SG infection. Once birds displayed severe lethargy, immobility, and greenish diarrhoea, they were culled and categorised under the mortality group for further analysis. All animals were handled following guidelines set by the Animal Ethics Committee (NON2023-135-001) in compliance with the Korean Council on Animal Care and the Korean Animal Protection Law, 2007: Article 13.

The attenuated strains, JOL3015 and JOL3016, were administered at concentrations of 1 × 10⁷ CFU/bird (Low) and 1 × 10⁸ CFU/bird (High) to evaluate bacterial persistence in vital organs. SG9R and SG WT JOL422 were administered at a concentration of 1 × 10⁷. Three chickens from each group were sacrificed at 3, 7, and 14 days post-inoculation (dpi) for sample collection. After the chickens were euthanised, the spleen and liver were aseptically collected, which were then homogenised in PBS using a mechanical homogeniser (IKA T 10 basic ULTRA-TURRAX, Germany) and plated on BGA at 10-fold serial dilutions to quantify the bacterial load.

Additionally, cloacal swabs were collected using a sterilised cotton swab in 1 mL of PBS to evaluate bacterial shedding for environmental safety. The swab samples were thoroughly mixed, serially diluted in PBS, and then plated on BGA. Changes in body weight were monitored every three days for up to 15 days after inoculation to determine how the infection affected weight gain.

### Histopathological evaluation of organ damage

Histopathological examination was undertaken on the liver, spleen, and cecum tissue sections using haematoxylin and eosin (H&E) staining. Three birds per group were sacrificed on the seventh day post-inoculation, with the organs collected and fixed in 10% formalin. The tissues were sectioned into 3 μm slices, fixed, and processed according to a standard protocol for H&E staining. This process involved dehydration, clearing, embedding, and staining to allow for clear visualisation of tissue architecture. A comprehensive investigation of potential tissue damage was performed using a Zeiss Axio Imager.M2 microscope (Carl Zeiss AB, Stockholm, Sweden). Microscopic examination was conducted to evaluate the cellular and structural integrity, and the resulting images were documented for further analysis.

### Immunisation and challenge against fowl typhoid using attenuated SG strain

The immune response elicited by inoculating 4-week-old female brown chickens with attenuated SG strains was evaluated. Each bird (*n* = 8) was intramuscularly immunised with JOL3015 and JOL3016 strains at a concentration of 1 × 10⁷ CFU/200 µL. A commercial vaccine strain, SG9R, was administered intramuscularly as a comparative control. Additional groups served as PBS and naïve controls. After two weeks, the birds received a booster immunisation with the attenuated SG strains. Serum and cloacal swab samples were collected at intervals for five weeks following the initial inoculation. The cloacal swabs were collected by inserting a sterile cotton swab into the cloaca and immediately breaking the swab section into 1 mL of PBS-containing tubes, which were stored at 4 °C during the collection. The samples were then stored at −80 °C until further analysis and later used to measure levels of IgY and IgA antibodies.

Furthermore, blood samples were collected two weeks after the booster immunisation, and PBMCs were isolated. T-cell counts were assessed using flow cytometry to quantify the cell-mediated immune response. Three weeks after booster application, the chickens were challenged with SG WT JOL422 via the oral route using 1 × 10^6^ CFU/200 µL per bird. The post-challenge survival rate was evaluated by monitoring for up to 15 days. Birds displaying severe lethargy, immobility, and greenish diarrhoea were culled and categorised under the mortality group for further analysis. Bacterial persistence in the spleen and liver of immunised chickens was investigated one week after the challenge to elucidate the bacterial load. In addition, the spleen and liver tissues were collected for H&E staining as described elsewhere [20]. At the end of the experiment, animals were sacrificed to examine any gross morphological distortions in their vital organs.

### ELISA for cytokines quantification and humoral and mucosal immune responses

Chickens inoculated with engineered SG strains underwent endotoxicity assessment by quantifying inflammatory cytokines, with serum samples collected on day three post-inoculation. The levels of inflammatory cytokines, including TNF-α, IL-1β, and IFN-γ, were measured using commercial sandwich-ELISA kits following the manufacturer’s instructions. Briefly, micro-ELISA plates pre-coated with an antibody specific to chicken TNF-α (Cat. No. MBS2509660, MyBioSource, San Diego, USA), IL-1β (Cat. No. MBS2702032, MyBioSource) and IFN-γ (Cat. No. MBS2700893, MyBioSource) were incubated separately with serum samples along with corresponding standards. A biotinylated detection antibody and an avidin-horseradish peroxidase (HRP) conjugate were successively added to the microplate wells and incubated. After washing, a substrate solution was added to initiate the enzyme reaction, which was then halted with a stop solution. The Infinite M200 spectrophotometer (Tecan) was used to measure the optical density (OD) at 450 nm. Cytokine concentrations were estimated using a reference standard.

*Salmonella*-specific systemic IgY and mucosal IgA responses in immunised birds were quantified using an indirect ELISA. Plates were coated with 400 ng/well of crude SG WT strain protein in carbonate-bicarbonate buffer and incubated overnight at 4 °C. Blocking was done with 5% skim milk. Serum samples were diluted 1:50 for IgY detection, while undiluted cloacal swab samples were used for IgA detection. Samples were added to the wells and incubated for 2 h at room temperature (RT), followed by incubation with goat anti-chicken IgY-HRP (Bethyl Laboratories, Texas, USA) or goat anti-chicken IgA-HRP (Bethyl Laboratories, Texas, USA) according to the manufacturer’s instructions. After washing, the O-phenylenediamine dihydrochloride substrate (Sigma, Missouri, USA) was added for colourimetric detection. The enzyme-substrate reaction was stopped with 50 µL of 2 N sulfuric acid, and OD at 492 nm was measured using an Infinite M200 microplate reader (Tecan). The obtained absorbance values were used to quantify the levels of IgY and IgA antibodies in the serum and cloacal swab samples, respectively.

### Flow cytometry

The cell-mediated immune responses were investigated by evaluating T-lymphocyte subsets using flow cytometry analysis. Two weeks after administering the booster immunisation, blood was collected from all groups (*n* = 5) to isolate PBMCs. Briefly, blood was diluted 1:1 with PBS (pH 7.4) and carefully layered over an equal volume of Ficoll-Paque PLUS density gradient media. Samples were centrifuged at 400 × *g* for 30 min at 18 °C, and PBMCs separated at interface layers were collected. Cells were then resuspended in RPMI-1640 medium supplemented with 10% FBS and 1% antibiotics and seeded in 96-well plates at 1 × 10^5^ cells/well. The cells were stimulated with 400 ng/well of crude soluble antigen extracted from the SG WT strain for 72 h in a 5% CO_2_ incubator at 37 °C.

Cells were collected and incubated with fluorescently labelled antibodies: anti-CD3-FITC (Clone CT-3, Cat: 8200-02, SouthernBiotech, Birmingham, AL, USA), anti-CD8a-PE (Clone CT-8, Cat: 8220-09, SouthernBiotech), and anti-CD4-AF700 (Clone CT-4, Cat: 8210-27, SouthernBiotech) (each at a concentration of 8 µg/mL) at 4 °C for 30 min in the dark. After incubation, the cells were washed with FACS buffer (PBS containing 2% FBS and 0.1% sodium azide) to remove unbound antibodies. The stained cells were then analysed with 1 × 10^4^ cells/sample acquisition using a MACSQuant flow cytometer (Miltenyi Biotec, Bergisch Gladbach, Germany). As a gating strategy, first, total lymphocytes were selected, and CD3^+^ cells were gated, from which CD3^+^CD4^+^ and CD3^+^CD4^+^ cells were quantified using non-stained cells and fluorescence minus one control. The results were analysed using MACSQuant analysis software (version 2.6), allowing for a detailed assessment of the cell-mediated immune response elicited by the immunisation.

### Statistical analysis

Statistical analysis was performed using Student’s *t*-test and ANOVA to evaluate statistical differences. A *p*-value < 0.05 was considered significant. All analyses were done in GraphPad Prism 9.00 software (San Diego, CA, USA).

## Results

### Development of attenuated SG strain

**Figure 1:**
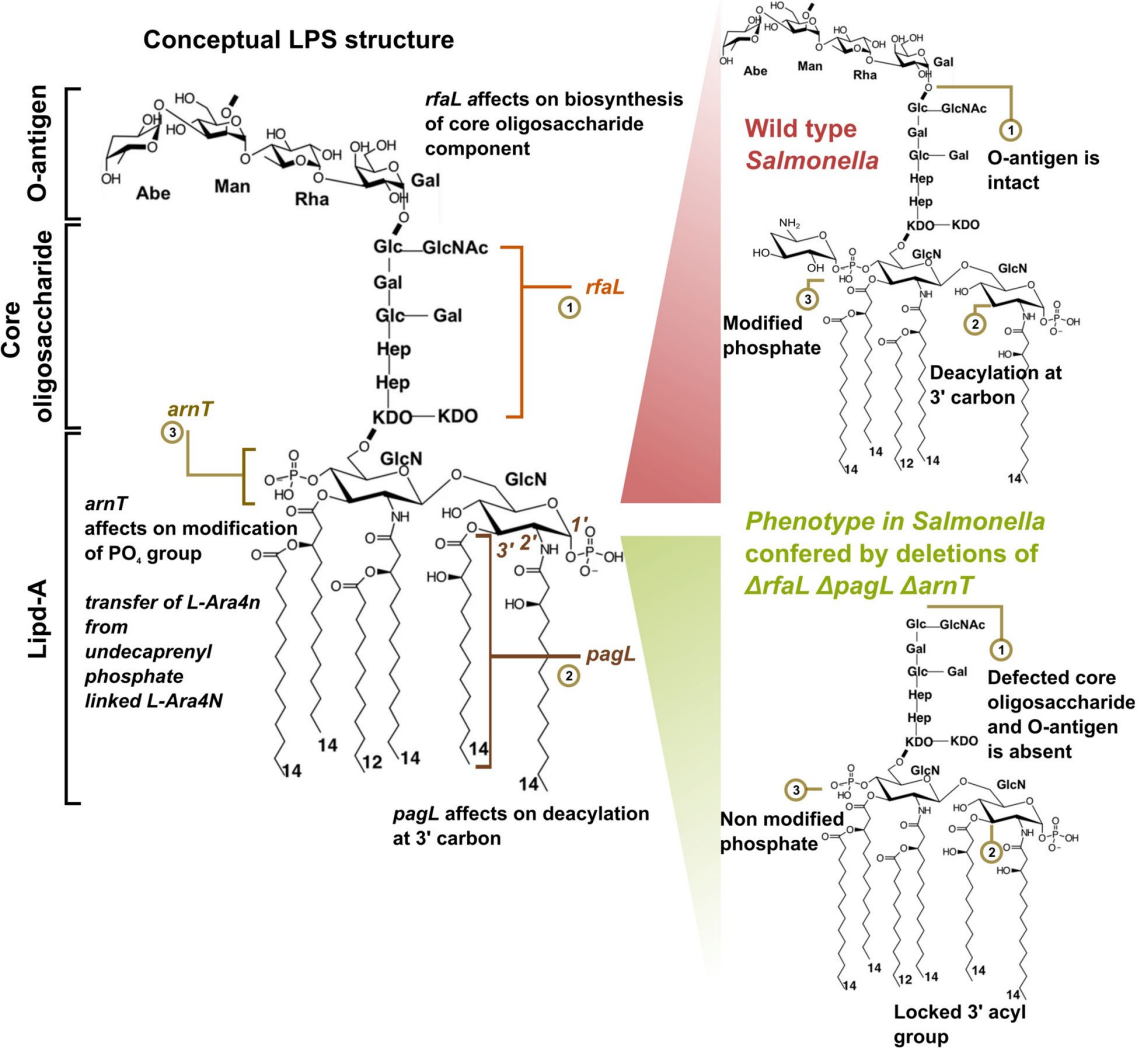
**Structural modifications of lipopolysaccharide (LPS) resulting from gene deletions.** The schematic representation illustrates the structural components of LPS in wild-type and genetically modified strains. The non-modified LPS comprises three main components: Lipid A, Core oligosaccharide, and O-antigen. In the genetically modified strain, the *rfaL* gene deletion results in the absence of the O-antigen. Additionally, *pagL* gene deletion leads to the lack of deacylated Lipid A. Furthermore, the *arnT* gene deletion prevents the addition of 4-amino-4-deoxy-l-arabinose (l-Ara4N) to the phosphate groups of Lipid A. These gene deletions result in significant structural modifications of the LPS, which are critical for understanding the functional and immunogenic implications of bacterial pathogenesis.

The SG strains were engineered to possess defective LPS structures using the well-established lambda red recombination method [21]. This recombineering approach involved replacing the selected genes with a flippase recognition target (FRT) flanked chloramphenicol resistance (*catR*) gene in the chromosome. The targeted deletions included four specific genes: *lon*, *rfaL*, *pagL*, and *arnT*. Confirmation of these deletions was achieved through flanking PCR [15], as depicted in Additional file 1 (PCR results), using specific flanking primers listed in Table 2. Notably, deleting *rfaL* impacted the biosynthesis of the core oligosaccharide, resulting in modified LPS lacking O-antigen attachment. The *arnT* deletion supposedly alters the transfer of L-Ara4N to the phosphate group, affecting the overall charge of the cell surface. Deleting pagL may block lipid A’s deacylation, preventing further modifications in the LPS structure. The conceptual framework of these deletions is depicted in Figure 1.

### Phenotypic and biological characterisation

**Figure 2:**
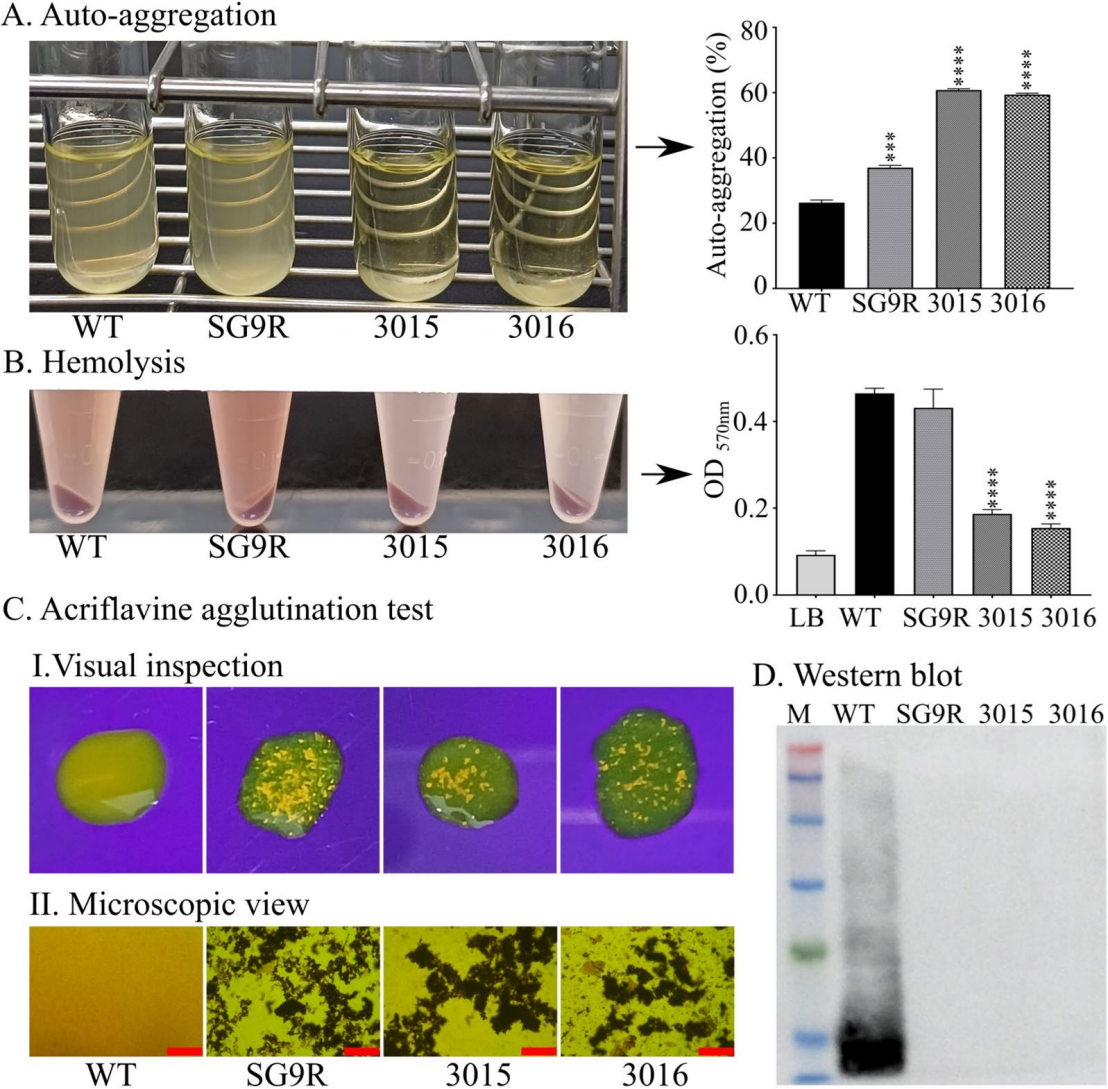
**Phenotypic and biological characterisation of *Salmonella *Gallinarum strains**. **A** Auto-aggregation. Visual observation of auto-aggregation in bacterial cultures grown statically at 37 °C for 24 h. The percentage of auto-aggregation was calculated by comparing the OD_600_ values from the upper layer of the culture with those from the resuspended culture after vortexing. **B** Haemolytic Activity. Haemolytic activity was assessed using the supernatant from mutant bacterial cultures incubated with a 10% chicken red blood cell (RBC) suspension at a 4:1 dilution for 12 h at 37 °C. Haemolytic activity was quantified by measuring the OD_570_ and comparing the mutant strains to the wild-type. Statistical analysis was performed using one-way ANOVA, with data presented as ***p < 0.001 and **** *p* < 0.0001. **C** Acriflavine Agglutination Test. The rough surface phenotype of mutant strains was confirmed by agglutination formation with acriflavine. Agglutination was observed under a microscope at 40× magnification. The scale bar represents 500 μm. **D** Western Blot Analysis of LPS. Lipopolysaccharide (LPS) was extracted from individual strains and analysed with a Western blot. The LPS was probed with a mouse antibody against *Salmonella* O antigen (primary antibody) followed by a goat anti-mouse IgG-HRP (secondary antibody). M denotes the protein molecular weight marker.

Our study found that bacteria demonstrate self-aggregation when cultured and that the hydrophobicity of their cell surfaces may influence this behaviour. Notably, mutant strains JOL3015 and JOL3016 exhibited significantly higher auto-aggregation abilities, with rates of 61% and 59%, respectively, compared to WT and SG9R strains, which had auto-aggregation rates of only 25% and 37%, respectively (Figure 2A). Moreover, the haemolytic assay revealed a remarkable reduction in hemolysis exceeding 50% in both mutant strains compared to the control (Figure 2B), indicating a significant alteration in their haemolytic properties. Additionally, the acriflavine agglutination test demonstrated agglutination in the presence of acriflavine for both mutant strains, suggesting a rough surface phenotype (Figure 2C). This attribute ensures that the lipid A core is exposed, allowing acriflavine to interact, thus leading to agglutination. When visualised under ultraviolet light, clear agglutination patterns were evident to the naked eye. Further analysis by western blotting confirmed the absence of interaction between the mutant strains and antibodies against *Salmonella* O-antigen, highlighting a phenotypic change induced by the LPS mutation in these strains (Figure 2D). These findings collectively underscore the influence of hydrophobicity and LPS modifications on the cell surface properties of these bacterial strains, providing valuable insights into their phenotypic characteristics.

### Bacterial growth kinetics of attenuated SG strains

**Figure 3:**
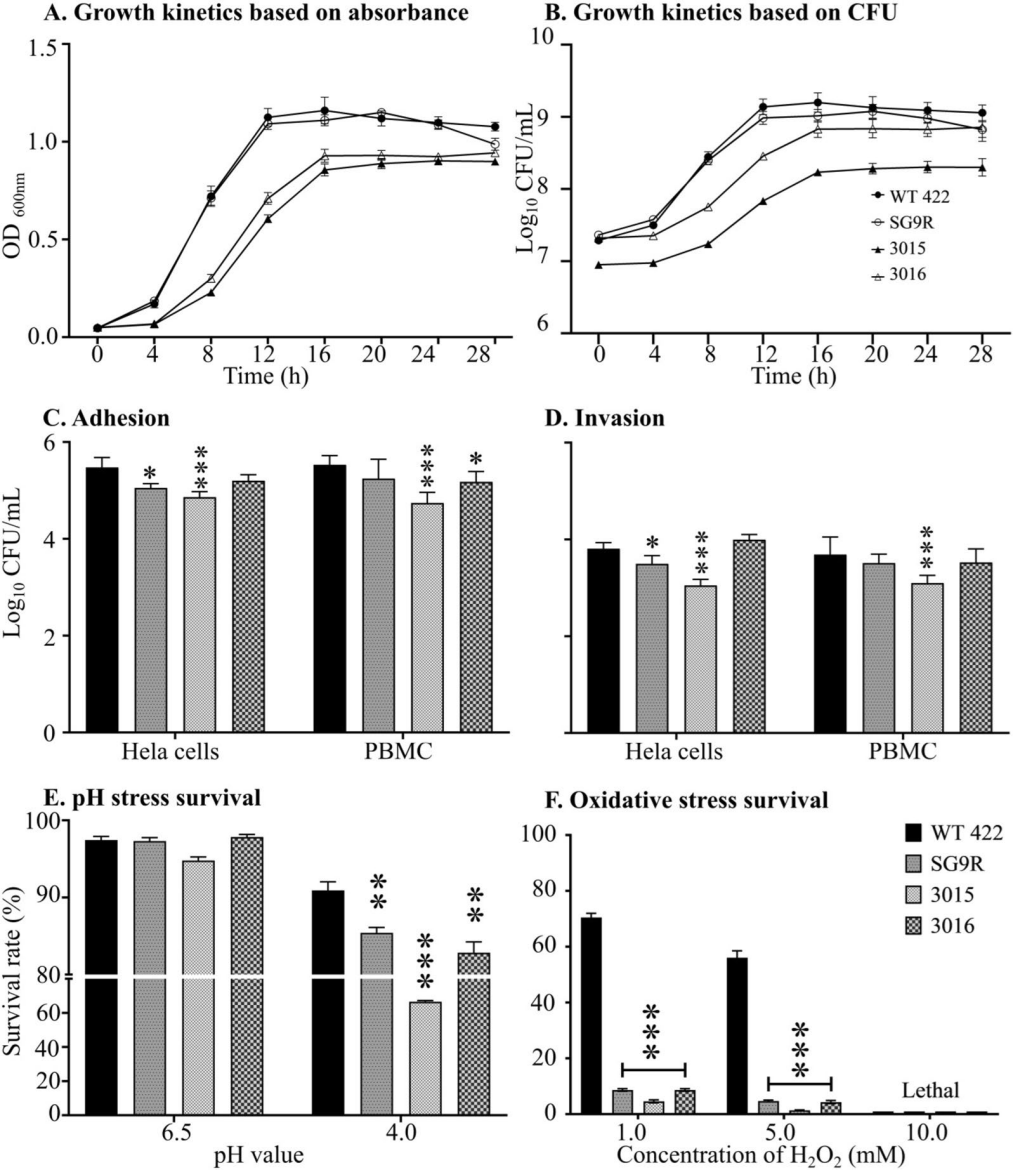
**Characterisation of attenuated *Salmonella *Gallinarum strains**. **A** Growth Curve Based on Absorbance. Growth kinetics were evaluated by measuring the optical density (OD) at 600 nm over time. **B** Growth Curve Based on CFU. Bacterial growth kinetics were assessed by plating cultures at respective time points at different dilutions on BGA media. The colony-forming units (CFU) per mL were then evaluated. **C** In Vitro Adhesion. The adhesion strengths of JOL3015, JOL3016, and SG9R strains were compared to the *Salmonella* JOL422 wild-type (WT) strain using HeLa cells and peripheral blood mononuclear cells (PBMCs). Monolayer cells were infected with each strain at a multiplicity of infection (MOI) of 40. Adhesion was assessed after 30 min of incubation. **D** In Vitro Invasion. The invasion capacities of JOL3015, JOL3016, and SG9R strains were compared to the WT strain using HeLa cells and PBMCs. Monolayer cells were infected with each strain at an MOI of 40. Invasion was assessed after 2.5 h of incubation.** E** pH stress survival and (**F**) Oxidative stress survival. The survival of bacterial strains after stress was evaluated relative to their initial inoculum concentration. Data were analysed by multiple unpaired t-tests and are presented as *p < 0.05, **p < 0.01, and ***p < 0.001.

The growth kinetics of attenuated SG strains were evaluated and compared with the wild-type strain, JOL422, and a commercial strain, SG9R (Figure 3A, B). Throughout the experiment, discernible differences in growth dynamics were observed between the engineered SG strains and the wild-type counterpart. While the wild-type JOL422 and SG9R strains exhibited analogous growth patterns, significant disparities were noted with the engineered strains, particularly JOL3016 and JOL3015. During the initial growth phase, both JOL3015 and JOL3016 maintained a conspicuous gap compared to the wild-type strain, with JOL3016 displaying a slightly narrower gap than JOL3015. This disparity persisted up to 16 h of incubation, after which the gap gradually decreased and plateaued. Notably, the optical density at 600 nm (OD_600_) peaked between 16 and 20 h for the wild-type and SG9R strains, followed by a decline.

In contrast, JOL3015 and JOL3016 exhibited incremental bacterial growth up to 28 h. At 8 h, the wild-type strain demonstrated a 3.14 and 2.39-fold increase in OD_600_ compared to JOL3015 and JOL3016, respectively, narrowing to 1.35 and 1.25-fold at 16 h. The logarithmic phase was observed in all four strains between 4 and 12 h, with both absorbance and CFU increments being increased, thus narrowing the gap against the WT SG 422 at 28 h post-incubation. At the end of the incubation period, there was a minimal disparity in CFU growth between JOL3016 and SG9R, suggesting comparable growth kinetics.

### In vitro characterisation of bacterial virulence and environmental stress

The assessment of adhesion and invasion using Hela and chicken PBMC revealed JOL3016 with comparable results against the SG WT 422 strain. The adhesion (Figure 3C) and invasion (Figure 3D) capabilities of SG9R and JOL3015 were significantly lower than those of the GS WT 422 strain and JOL3016. The exposure of bacterial cells to acidic environments at 6.5 pH and 4.0 pH revealed that all strains could tolerate mild acid conditions at 6.5 pH. However, the increase in acidity at 4.0 pH showed that the mutants are susceptible to acidity. JOL3015 presented the lowest tolerance, while JOL3016 was comparable to the SG9R vaccine strain (Figure 3E). Furthermore, oxidative stress conditions induced by variable concentrations of H_2_O_2_ (mM) demonstrated a significant growth suppression even at 1.0 mM. At 5.0 mM concentration, bacterial cell growth was still present. However, 10.0 mM concentration was lethal to all bacterial strains (Figure 3F).

### In vitro assessment of cytotoxic responses

**Figure 4:**
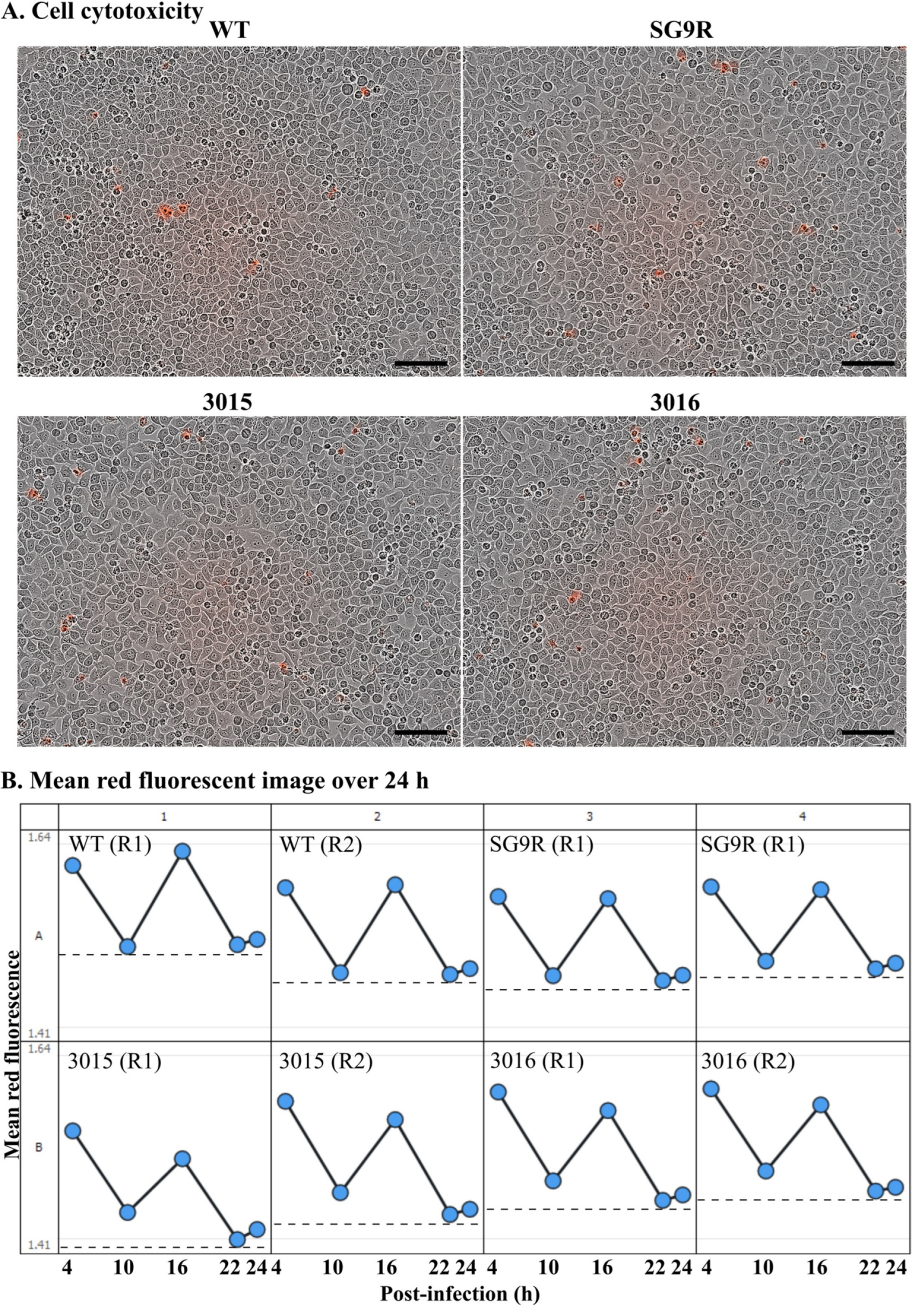
**Assessment of cell cytotoxicity.** **A** Cell Survival Assay. The attenuation level and persistence of mutant SG strains were evaluated using a cell survival assay. A confluent monolayer of HeLa cells was infected with wild-type (WT) JOL422, SG9R, JOL3015, and JOL3016 strains at a multiplicity of infection (MOI) of 40. Cell survival was monitored using propidium iodide staining, and cytotoxicity was assessed by real-time observation with the IncuCyte live imaging system over 24 h. Micrographs show images captured 24 h post-infection, with the scale bar representing 100 μm. **B** Cytotoxicity Observation. Higher retention of red-coloured objects was observed in WT-infected cells over the 24 h, indicating increased cytotoxicity. The dotted lines indicate the lowest mean fluorescence intensity. The experiment was repeated three times, with R1 and R2 representing the first and second replicates.

Intracellular cytotoxicity induced by each strain SG WT422, SG9R, JOL3015, and JOL3016 was investigated using the propidium iodide staining method. Cells were observed in real-time using the IncuCyte (Essen Bioscience, Gottingen, Germany) live imaging system (Figure 4A). Visual observation over 24 h showed the highest number of red fluorescing objects in cells treated with SG WT 422 strain. Furthermore, the matric quantification of mean red-fluorescent objects revealed that both SG9R and JOL3016 were comparable, while JOL3015 remained lowest in cytotoxic responses (Figure 4B).

### Safety assessment of the detoxified strains

The bacterial load in vital organs, including the spleen and liver, as well as in cloacal swabs, was evaluated to estimate the burden caused by the detoxified SG strains. Chickens were inoculated with mutant strains at two doses, 1 × 10^7^ and 1 × 10^8^ CFU/bird, via the intramuscular (IM) route and monitored over 15 days. Birds inoculated with the WT strain JOL422, which served as the control, displayed lethargic behaviour, which was characterised by depression, anorexia, ruffled feathers, diarrhoea, dehydration, and weight loss. In contrast, chickens from the other groups exhibited normal behaviour with usual feed and water intake. They also did not show adverse signs of inoculation or clinical symptoms, such as increased body temperature.

**Figure 5:**
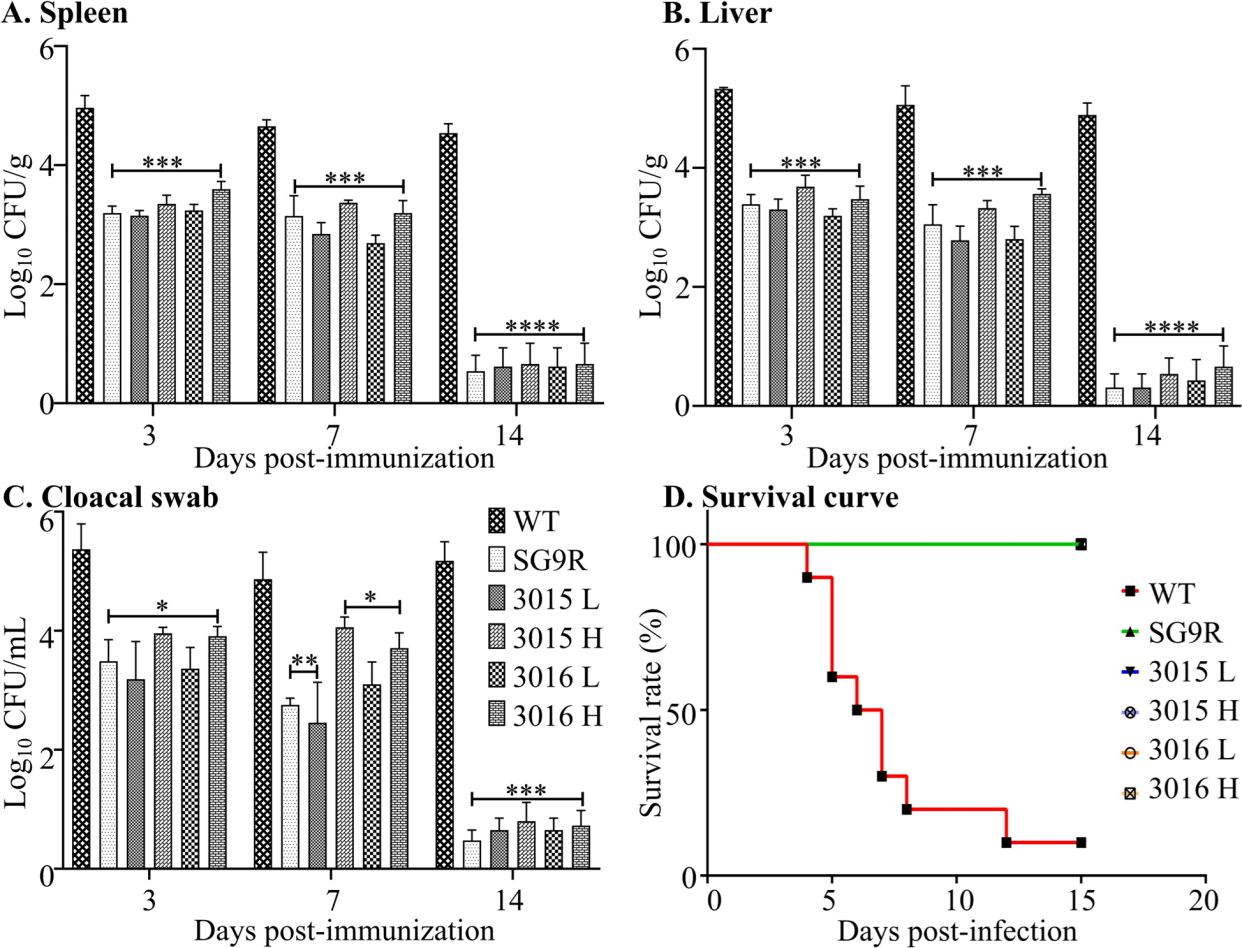
**Safety assessment of the attenuated strains.** **A**–**C** Bacterial localisation. Birds were inoculated intramuscularly with the developed strains JOL3015 and JOL3016 at 1 × 10^7^ CFU/bird (low dose, L) and 1 × 10^8^ CFU/bird (high dose, H) to evaluate the safety profile. Bacterial load was enumerated in the spleen (**A**), liver (**B**), and cloacal swabs (**C**). Data were analysed by multiple unpaired t-tests and are presented as *p < 0.05, **p < 0.01, and ***p < 0.001. **D** Kaplan-Meier Survival Curve. The survival of birds was monitored for 15 days post-inoculation to assess the safety of the strains. The Kaplan-Meier survival curve represents the percentage of surviving birds over the observation period.

The bacterial load in the spleen, liver, and cloacal swabs indicated the dispersal of bacteria throughout all tested organs and sites. Over time, the bacteria were gradually eliminated from their respective sites, with bacterial persistence lasting for 14 days, which assured the production of an immune response (Figure 5A–C). Bacterial retention of the attenuated strains inoculated at 1 × 10^7^ and 1 × 10^8^ CFU/bird in the selected lymphoid organs was comparable to that of SG9R injected at 1 × 10^7^ CFU/bird. Administration of a tenfold higher bacterial concentration, comparable to SG9R, demonstrated a safe response. As a positive control, WG WT 422 infection displayed more than 90% mortality within 5 to 15 days post-infection (Figure 5D). Overall, the results indicate reduced infectivity in both attenuated strains, while they retained desirable infectivity to induce immunogenicity.

**Figure 6:**
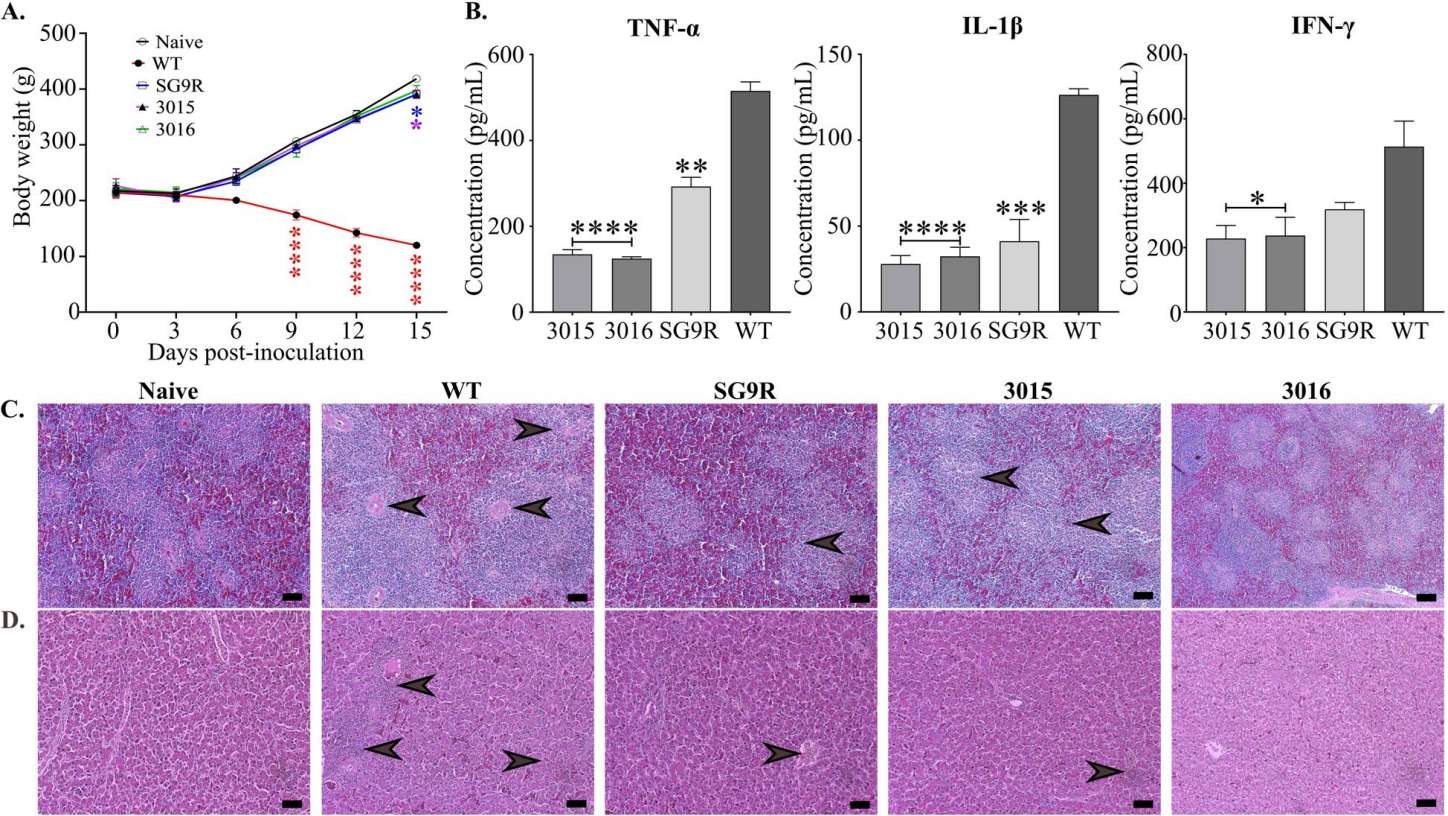
**Evaluation of safety and pro-inflammatory cytokines.** **A** Change in Body Weight. The change in body weight of chickens was monitored following the introduction of *Salmonella* Gallinarum (SG) strains. **B** Serum Cytokine Concentration. The concentration of pro-inflammatory cytokines in the serum was measured. Data were analysed by multiple unpaired t-tests and are presented as *p < 0.05, **p < 0.01, ***p < 0.001, and ****p < 0.0001. **C** Histopathological Evaluation of Spleen. Inflammatory response in the spleen of immunised birds was assessed. Significant tissue alteration, including degeneration and necrosis in the white pulp, was noted in chickens inoculated with the WT SG JOL422 strain (indicated by arrows). **D** Histopathological Evaluation of Liver. Inflammatory response in the liver of immunised birds was evaluated. The black arrow indicates the infiltration of immune cells in the liver of birds infected with the WT strain. The scale bar: 50 μm.

The introduction of SG as a live vaccine resulted in a mild decrease in body weight until the third post-inoculation day. Birds vaccinated with the commercial SG9R vaccine exhibited more than 7% body weight loss (Figure 6A). In contrast, birds inoculated with JOL3016 lost less than 5% body weight within 15 days compared to the naïve group. Modifying the LPS structure in both specially designed SG strains helped to address endotoxicity, which is a significant challenge in implementing live bacterial vaccines. The endotoxicity induced by these strains was corroborated by measuring inflammatory cytokines using sandwich-ELISA. The concentration of TNF-α, a major inflammatory cytokine marker, showed a significant reduction. For instance, JOL3015 and JOL3016 exhibited 3.82- and 4.13-fold decreases, respectively, while SG9R showed a 1.76-fold reduction compared to the WT (Figure 6B).

Notably, JOL3015 and JOL3016 induced 2.17- and 2.34-fold lower TNF-α production than the commercial SG9R strain. This outcome underscores the significance of the developed strains. Additionally, the production of IL-1β was down-regulated by 4.52- and 3.90-fold in the JOL3015 and JOL3016 groups (Figure 6B), respectively, compared to WT, which was 1.47- and 1.27-fold lower than SG9R. Furthermore, the endotoxicity-related pro-inflammatory cytokine IFN-γ showed elevated levels in the WT group compared to the other groups. Both developed strains demonstrated a down-regulation of IFN-γ by more than 2-fold (Figure 6B).

Histopathological examinations of H&E stained spleen and liver tissues also revealed the degree of damage SG WT 422 strain induced in spleen and liver tissues. Analysis showed expanded white pulp areas of lymphatic tissues in the spleen and signs of severe inflammation and potentially necrotic regions in liver tissues. Compared to the WT inoculation group, all vaccinated groups demonstrated lower levels of tissue damage, particularly for the SG9R and JOL3016 strains (Figure 6C, D). This investigation indicates that our strains can induce lower endotoxicity than the WT group. This outcome highlights the potential of these developed strains in minimising inflammatory responses. These findings support the notion that the engineered strains JOL3015 and JOL3016 are safe and effective in eliciting an immune response without the adverse effects typically associated with live bacterial vaccines.

### Humoral and mucosal immune response

**Figure 7:**
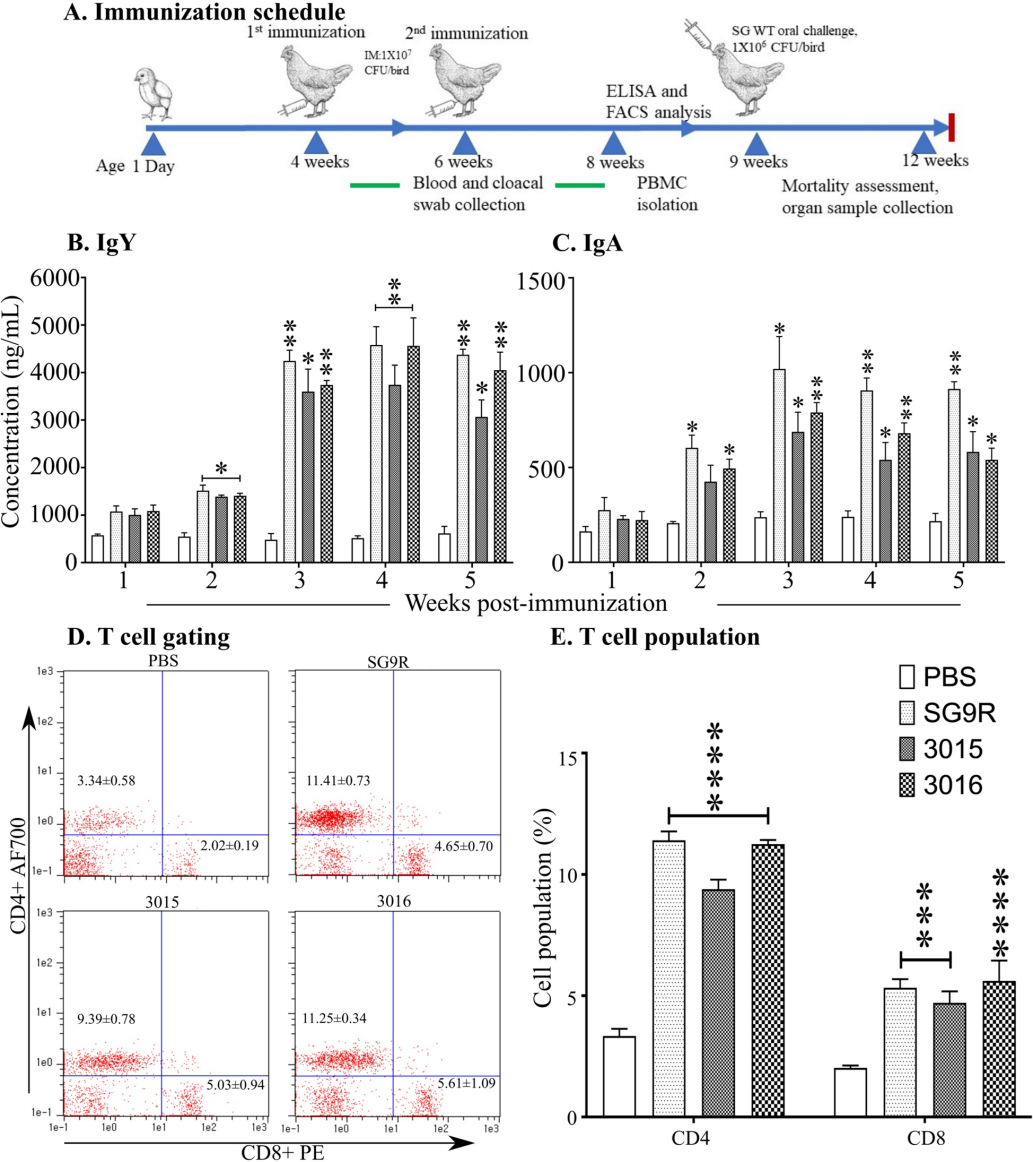
**Humoral and cellular immune response post-immunisation.** **A** Immunisation Schedule. Booster immunisation was administered in the second week following the initial immunisation, and samples were collected at respective points. **B** IgY Antibody Production. **C** IgA Antibody Production. IgY antibody production in serum and IgA in cloacal secretions in response to immunisation was assessed using indirect ELISA over five weeks post-immunisation. **D** Flow Cytometry Analysis. Representative flow cytometry scatter plots show the gating of CD4^+^ and CD8^+^ cells post-immunisation. **E** T-Cell Percentages. The histogram represents the percentage of CD4^+^ and CD8^+^ T cells in immunised birds. Data were analysed by multiple unpaired t-tests, with significant differences presented as *p < 0.05, **p < 0.01, and ***p < 0.001 compared to the PBS control.

Assessment of humoral immune responses upon immunisation demonstrated an increase in IgY (Figure 7A, B) levels in blood and sIgA (Figure 7C) in mucosal swabs. The response of IgY almost doubled after receiving the booster immunisation. Notably, the immune responses derived by SG9R and JOL3016 were comparable in the third, fourth, and fifth weeks of post-primary inoculation. In contrast, JOL3015 derived slightly lower IgY responses than SG9R and JOL3016. Peak IgY responses resulted in three weeks of post-priming and were sustained until the fifth week post-priming. The sIgA responses also peaked at three weeks post-priming and sustained until the fourth week of post-priming. A significant increase in sIgA responses was noted on booster immunisation, and JOL3016 was comparable to the SG9R vaccine strain.

### Cell-mediated immune responses

The cell-mediated immune response elicited by immunisation was evaluated by quantifying T-cell populations using flow cytometry analysis. The primary focus was to differentiate between the T-lymphocyte subsets, specifically CD4^+^ and CD8^+^ T cells, within PBMCs. Flow cytometric analysis showed a significant increase in CD3^+^CD4^+^ and CD3^+^CD8^+^ T-cell populations in the immunised chickens, indicating an enhanced cell-mediated immune response (Figure 7D, E) (for gating strategy, Additional file 2). Chickens immunised with the SG JOL3016 strain exhibited a notable rise in CD4^+^ and CD8^+^ T cells (Figure 7D, E), comparable to the immune response observed with the commercial vaccine strain SG9R. The CD3^+^CD4^+^ and CD3^+^CD8^+^ T-cell populations for SG9R were 11.40% and 5.32%, respectively. However, for JOL3016, these populations were 11.25% and 5.61%. These results indicate that immunisation with the SG JOL3016 and JOL3015 strains significantly increases CD4^+^ and CD8^+^ T cells, comparable to the response induced by the commercial SG9R vaccine strain. These findings demonstrate the potential of the engineered strains to elicit a robust cell-mediated immune response, which is crucial for effective immunoprotection.

### Protection against wild-type challenge

**Figure 8:**
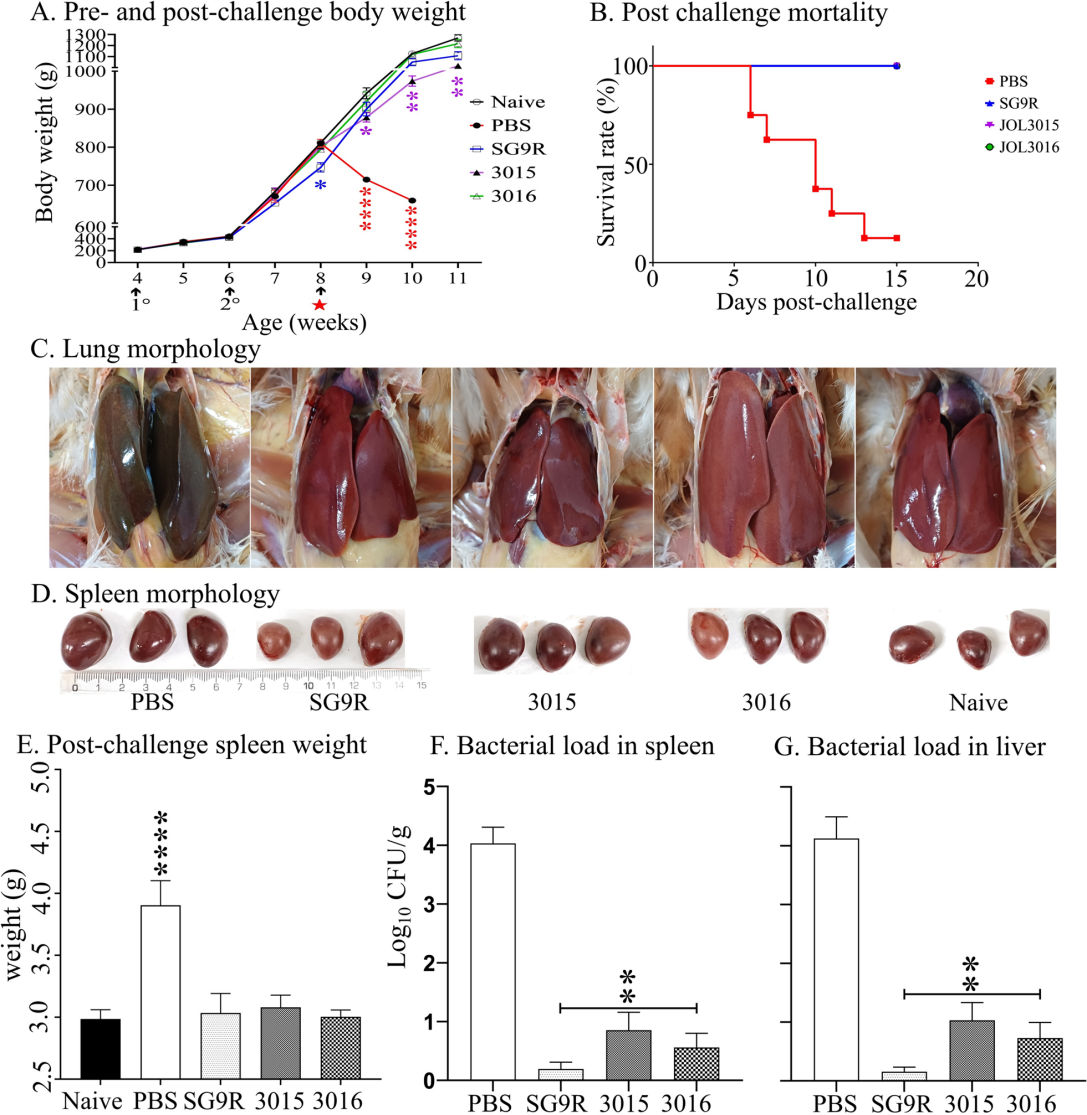
**Evaluation of immunised chicken upon challenge**. **A** Body Weight Alteration. Changes in chicken body weight were recorded pre- and post-challenge to assess the effect of immunisation on overall health and the degree of protection against the wild-type challenge. **B** Survival Rate. The survival of immunised birds challenged with the wild-type strain was compared with that of the non-immunised group. A Kaplan-Meier survival curve was developed using mortality records over 15 days post-challenge. **C** Liver Morphology. Morphological changes in the liver were examined for hepatic lesions post-challenge. **D** Spleen Morphology. The spleen was examined for splenomegaly and other morphological changes post-challenge. **E** Spleen Weight. Post-challenge spleen weights were measured and compared with those of naïve birds. The bacterial load of the wild-type challenge strain was assessed at 7 days post-infection for (**F**) Spleen. **G** Liver. Data were analysed by multiple unpaired t-tests, with significant differences from the PBS control presented as *p < 0.05, **p < 0.01, and ***p < 0.001.

Following the designated vaccination schedule, the chickens were immunised and then exposed to the SG WT 422 strain through intramuscular (IM) injection. Body weight measurements and observations for potential mortality were conducted regularly throughout the experiment. Immunisation with detoxified SG strains did not induce adverse reactions during the study period. The effect of detoxified SG strains on the weight gain of chickens was especially noticeable during the sixth to ninth weeks, displaying a higher increase in weight compared to the SG9R vaccine strain. Specifically, chickens immunised with the JOL3016 strain demonstrated body weight gains comparable to the naïve group (Figure 8A). Upon challenge, chickens in the PBS group experienced severe weight reduction and mortality due to SG infection, whereas all the immunised birds were protected against the lethal challenge (Figure 8B). Furthermore, the PBS group exhibited increased body temperature (Additional file 3), while the other groups demonstrated only marginal changes.

Further evaluations revealed that liver morphology (Figure 8C) and splenomegaly (Figure 8D) in immunised groups corroborated the levels of protection provided by both SG9R and JOL3016 detoxified strains. Post-challenge assessments showed significant yet comparable outcomes in spleen weight and bacterial loads found in spleen and liver tissues between the SG9R and JOL3016 immunised groups (Figure 8E–G). The bacterial load in the PBS group was around log_4_ CFU/g, whereas the loads in SG9R and JOL3016 immunised groups were reduced to less than log_1_ CFU/g. Immunisation with detoxified SG strains, particularly JOL3016, prevented adverse reactions, promoted significant weight gain, and provided robust protection against lethal challenges. These findings highlight the potential of detoxified SG strains in effectively safeguarding against SG infection while supporting healthy growth in chickens.

### Histopathological examination

**Figure 9:**
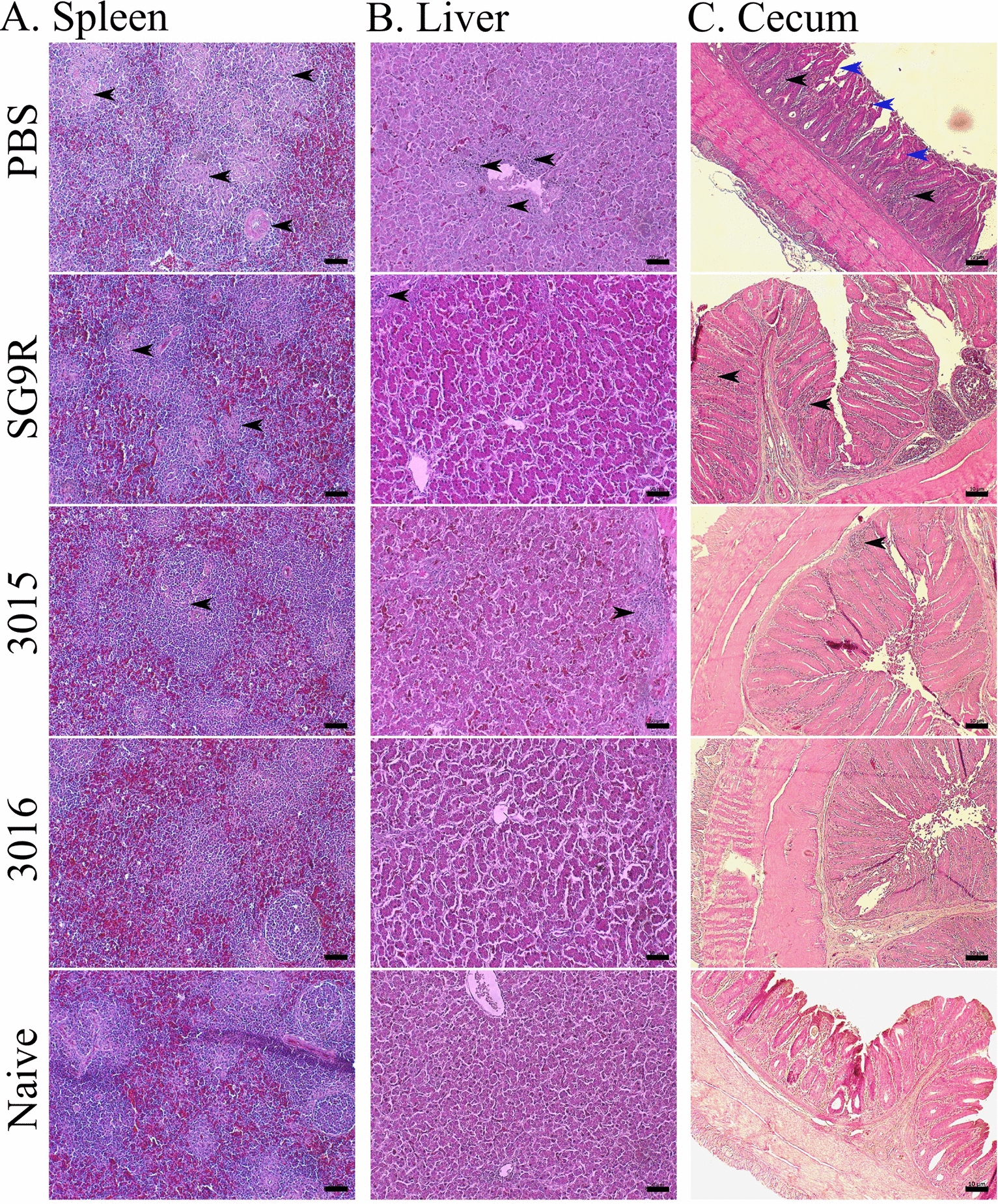
**Histopathological changes and microscopic lesions in chickens orally infected with the wild-type strain.** Chickens were orally infected with 1 × 10^6^ CFU/bird of *Salmonella* Gallinarum wild-type strain. Histopathological analysis of the internal organs was performed using H&E staining. **A** Spleen. Altered cellular alignment and tissue architecture were visualised in the spleen tissues (200×). In the PBS control group, degeneration and necrosis in the white pulp were observed, indicated by arrows. **B** Liver. Altered tissue architecture and inflammatory lesions characterised by marked infiltration of heterophils and lymphocytes with degeneration and necrosis were observed in the liver tissues (200×). Arrows highlight inflammatory lesions in the liver. **C** Cecum. Tissue disturbance in the cecum with thickened and shortened villi structures was noted in the PBS group compared to vaccinated groups (40×). Black arrows denote immune cell infiltration, and blue arrows indicate the shortening and congestion of villi. The organs of uninfected chickens (naïve) were used as the control. Data were visualised and analysed using light microscopy. The scale bar: 50 μm for spleen and liver, and 10 μm for cecum.

A histopathological evaluation of the spleen, liver, and cecum tissues (Figure 9A–C) was conducted one week after an oral challenge with the SG WT 422 strain. The spleen tissues of naïve birds, the white pulp (lymphatic tissues) and the red pulp (venous sinuses) were clearly differentiated. Immunised birds with JOL3016, JOL3015, and SG9R strains showed substantial preservation of this tissue architecture. In contrast, the PBS control group exhibited a markedly expanded white pulp, indicating severe infection and inflammation (Figure 9A). In the liver tissues of the PBS group, severe necrotic discolourations were evident, reflecting extensive tissue damage. Liver tissues from immunised birds were comparable to those of the naïve group, although infiltration of Kupffer cells was observed across all groups, suggesting an active but controlled immune response (Figure 9B).

Analysis of the cecum tissues via histopathology showed significant erosion, crypt abscesses, and signs of oedema in the PBS group, indicating a severe bacterial infection. In contrast, immunised chickens showed considerable protection whether vaccinated with detoxified SG strains or the SG9R vaccine strain. Their cecum tissues were largely free from these severe pathological signs, demonstrating the effectiveness of immunisation in mitigating infection-induced tissue damage. These histopathological findings emphasise the protective efficacy of the JOL3016, JOL3015, and SG9R vaccine strains (Figure 9C). Compared to the non-immunised PBS group, immunised birds maintained a closer resemblance to naïve tissue architecture across vital organs, significantly reducing infection-related damage and inflammatory responses.

## Discussion

Fowl typhoid remains a significant concern in the poultry industry, particularly in developing regions where it inflicts substantial economic losses [22]. The causative agent, SG, not only impacts productivity but also poses risks to animal welfare and public health. The SG9R vaccine is widely used to mitigate the disease; however, concerns regarding its safety and efficacy remain prevalent. Our study aims to address these concerns by engineering SG strains that have been attenuated through targeted genetic modifications, thereby increasing the safety and effectiveness of the vaccine. While effective in many cases, the SG9R vaccine presents several limitations that hinder its widespread use and effectiveness. Concerns about potential reversion to virulence and endotoxicity raise questions about its long-term efficacy and safety [23, 24]. The risk of SG9R reversion during field outbreaks poses a significant challenge, highlighting the need for alternative vaccine candidates. Moreover, the residual pathogenicity of SG9R, particularly in immunocompromised hosts, underscores the urgency to develop safer vaccine options [25]. Given the pivotal role of LPS in the pathogenesis of SG and host immune responses [26], our study focused on modifying the structure of LPS to improve vaccine safety and immunogenicity. LPS is a key virulence factor and immunogen, making it an attractive target for vaccine development. By targeting the virulence genes and genes involved in LPS biosynthesis and modification, such as *lon*, *rfaL*, *pagL*, and *arnT*, we aimed to attenuate SG strains while preserving their immunogenicity.

The Lon protease functions as a global regulator of bacterial virulence. Therefore, its deletion could cause the overexpression of several invasion-related genes by promoting antigen presentation. The *rfaL* gene encodes O-antigen ligase, which is essential for properly attaching the O-antigen component to the lipid A core component. The lack of the *rfaL* gene confers a truncated version of the LPS structure, which has proven essential in providing DIVA capability [9]. The other two gene targets, *arnT* and *pagL*, play crucial roles in modifying lipid A, a component of LPS, thereby influencing bacterial virulence and host immune response [27–29]. The addition of L-Ara4N by ArnT changes the structure of lipid A, decreasing its negative charge and enhancing its bacterial resistance to host defences [30, 31]. Conversely, PagL-mediated deacylation reduces LPS hydrophobicity, potentially evading host immune detection [27, 32]. These modifications highlight the complex interplay between bacterial adaptation and host immune evasion strategies. Figure 1 represents the concept behind lipid A modification by our selected gene targets in the present study.

Our study employed a well-established lambda red recombineering approach to engineer attenuated SG strains with targeted in-frame deletions of *lon*, *rfaL*, *pagL*, and *arnT* genes (Additional file 1) in the SG genome. These deletions significantly modified the LPS structure, including changes in core oligosaccharides, O-antigen attachment, surface charge, and lipid A composition [9, 13, 15]. Importantly, these modifications aimed to reduce endotoxicity while maintaining vaccine efficacy. The engineered SG strains were characterised phenotypically and biologically, revealing altered surface properties (Figure 2A) and reduced haemolytic activity (Figure 2B). Truncation of the O-antigen component was confirmed by acriflavine agglutination assay (Figure 2C) and LPS Western blot (Figure 2D), which revealed a complete absence of the O-antigen component.

The modified LPS structure results in a rough surface that increases the hydrophobicity and causes the cells to aggregate and settle. Such modifications change the phenotypic features and affect biological characteristics, as evidenced by a decreased hemolysis activity. The significant decrease in hemolysis caused by the mutant strains indicates a reduction in virulence. This decrease needs to be considered when developing the vaccine strain, especially as the hemolysins of *Salmonella* play an essential role in intra-macrophage survival, killing cells, and prolonged systemic salmonellosis [33]. Moreover, examining bacterial growth kinetics offers insights into the differentiated physiological state of bacteria [34]. Our growth assessment in this study revealed distinctive growth kinetics compared to wild-type and commercial SG9R strains. The complete elimination of three genes from each detoxified SG strain, namely, *lon*, *rfaL*, and *arnT* from JOL 3015 and *lon*, *rfaL*, and *pagL* from JOL3016, resulted in a comparatively lower growth rate than the wild-type and SG9R vaccine strain at early time points of growth, however reducing the gap with an increase in incubation time (Figure 3A, B).

In particular, JOL3016 was almost equal in bacterial number to WT and SG9R within a 28 h incubation period, demonstrating that the strain was not overly attenuated. The selected genetic markers did not significantly affect bacterial adhesion or virulence, especially for the JOL3016 strain that carries *pagL* deletion. This outcome ensures that these strains retain their capability to invade host cells, which is essential for better antigen presentation (Figure 3C, D) [35]. Acidic and oxidative stress survival assays also revealed that JOL3016 is comparable to the SG9R vaccine strain. However, JOL3015 was found to have a slightly lower tolerance to acidity and oxidative conditions than the SG9R and JOL3016 (Figure 3E, F). These findings enable the detoxified SG strains to potentially undergo rapid clearance from the intracellular oxidative stress without persisting as a chronic infection, which may be an important safety consideration.

Further to note is that the mutant strains induced lowered cytotoxic responses without significantly damaging epithelial monolayers of Hela cells. The findings here also showed that JOL3016 was comparable to SG9R, while the lowest cytotoxic response was exhibited by the JOL3015 strain, exacerbating its stronger attenuation phenotype (Figure 4A, B). The safety assessments undertaken in the study also demonstrated minimal adverse reactions and reduced endotoxicity in inoculated chickens with detoxified strains. For example, no deaths occurred when birds were inoculated with detoxified SG strains or SG9R, while infection and mortality rates were significant when inoculated with the SG WT 422 strain. A comparison of two inoculation doses administered via the IM route, at 1 × 10^7^ and 1 × 10^8^ CFU/bird, was found to be completely safe for young chickens. Additionally, this treatment did not affect chicken growth to the same extent as SG9R.

It is worth noting that the examination of bacterial persistence in the spleen, liver, and cloacal swabs did not reveal any significant difference between the two inoculation doses (high and low). However, by day 14 post-inoculation, bacterial persistence had reduced to less than log 2 in all organ samples, spleen, liver, and cloacal swabs collected from challenged chicken. These findings underscore the safety and potential of the engineered strains as vaccine candidates (Figure 5). To further evaluate the reduced levels of endotoxicity responses, we investigated the levels of pro-inflammatory cytokines in blood samples. The results showed significantly lower levels of markers for pro-inflammatory cytokines, such as tumour necrosis factor-alpha (TNF-α), Interleukin-1β (IL-1β), and Interleukin-γ (IFN-γ), even lower than those in the SG9R vaccine strain (Figure 6). These observations were further exacerbated in the histopathological examination of spleen and liver tissues. The examination showed lowered signs of inflammation marked by red and white pulp distribution in the spleen and necrotic lesions, as well as severe inflammation in liver tissues.

The evaluation of humoral and cell-mediated immune responses showed that the engineered SG strains elicited a robust immune response comparable to the response elicited by the commercial vaccine strain SG9R. As live attenuated vaccine strains, chicken immunisation has resulted in a significant engagement of CD3^+^CD4^+^ and CD3^+^CD8^+^ differentiation (Figure 7D, E). CD3^+^CD4^+^ T cells also play a crucial role in activating macrophages and CD8^+^ T cells, ensuring a robust and coordinated immune response. Their role is pivotal in generating a strong humoral response, essential for neutralising pathogens and preventing infection spread. On the other hand, CD3^+^CD8^+^ T cells, known as cytotoxic T cells, are directly involved in eliminating infected cells. They recognise and kill cells presenting specific antigens on their surface, typically through the major histocompatibility complex class I (MHC I) pathway. This cytotoxic activity is essential for controlling intracellular pathogens such as SG by limiting bacterial replication and spreading within the host. Furthermore, CD8^+^ T cells produce various cytokines that contribute to the overall immune response and aid in the recruitment and activation of other immune cells.

The collective outcome and effectiveness of protective immune responses induced by novel vaccine candidates are clearly demonstrated in post-challenged pathological assessments. Importantly, post-challenge survival rates and histopathological analyses validate the protective efficacy of the engineered strains against wild-type SG challenge (Figures 8, 9). These results emphasise the potential of the engineered SG strains to induce protective immunity while minimising adverse reactions and pathological manifestations.

In conclusion, this study sheds light on the promising potential of engineered SG strains featuring modified LPS structures as safe and efficacious vaccine candidates against fowl typhoid. Notably, comparative analyses against the commercial vaccine strain SG9R underscored the superiority of the designed strains in terms of reduced endotoxicity and retained protective efficacy. These findings highlight the importance of further research to investigate the long-term efficacy and real-world application of the engineered strains in poultry populations.

## Supplementary Information

**Additional file 1. Confirmation of deletion of** ***lon*** , ***rfaL*** , ***pagL*** , **and** ***arnT*** **genes**. Flanking primers were used to confirm the deletion of respective genes. M = DNA marker, WT = Wild-type, and 1, 2, and 3 = Samples.

** Additional file 2. Gating strategy used for T-cell subsets , a representative sample for the JOL3016 group.** (A) Gating of Total lymphocytes. (B) Gating of CD3 + T cells from total lymphocytes. (C) Gating of CD3 + CD4 + and CD3 + CD + T cells from CD3 + T cells.

**Additional file 3. Measurement of body temperature (°C) at post-immunisation.**

## Data Availability

Raw data reported in the manuscript can be made available upon request from the corresponding author.

## Abbreviations

FT: fowl typhoid
LPS: llipopolysaccharide
SG: Salmonella enterica serovar Gallinarum (*Salmonella* Gallinarum)
pagL: PhoP/PhoQ-activated gene
arnT: l-Ara4N transferase gene
DIVA: differentiate infected from vaccinated animals
ELISA: enzyme-linked immunosorbent assay
catR: chloramphenicol resistance gene
OD: optical density
RBC: red blood cell
PBMCs: peripheral blood mononuclear cells
PBS: phosphate-buffered saline
WT: wild-type
dpi: days post-inoculation
H&E: haematoxylin and eosin staining
RT: room temperature
TNF-α: tumour necrosis factor-alpha
IL-1β: Interleukin-1β
IFN-γ: Interleukin-γ

## References

[1] Zhou X, Kang X, Zhou K, Yue M (2022) A global dataset for prevalence of *Salmonella* Gallinarum between 1945 and 2021. Sci Data 9:495 35963862 10.1038/s41597-022-01605-x PMC9376096

[2] Alves Batista DF, de Freitas Neto OC, Maria de Almeida A, Maboni G, de Carvalho TF, de Carvalho TP, Barrow PA, Berchieri AJ (2018) Evaluation of pathogenicity of *Salmonella* Gallinarum strains harbouring deletions in genes whose orthologues are conserved pseudogenes in *S.* Pullorum. PLoS One 13:e0200585 30028856 10.1371/journal.pone.0200585 PMC6054384

[3] Arora D, Kumar S, Jindal N, Narang G, Kapoor PK, Mahajan NK (2015) Prevalence and epidemiology of *Salmonella enterica* Serovar Gallinarum from poultry in some parts of Haryana, India. Vet World 8:1300–1304 27047033 10.14202/vetworld.2015.1300-1304 PMC4774741

[4] Wigley P, Hulme S, Powers C, Beal R, Smith A, Barrow P (2005) Oral infection with the *Salmonella enterica* Serovar Gallinarum 9R attenuated live vaccine as a model to characterise immunity to fowl typhoid in the chicken. BMC Vet Res 1:2 16221297 10.1186/1746-6148-1-2 PMC1236940

[5] Lee YJ, Mo IP, Kang MS (2007) Protective efficacy of live *Salmonella* gallinarum 9R vaccine in commercial layer flocks. Avian Pathol 36:495–498 17994329 10.1080/03079450701691278

[6] Kwon YK, Kim A, Kang MS, Her M, Jung BY, Lee KM, Jeong W, An BK, Kwon JH (2010) Prevalence and characterization of *Salmonella* Gallinarum in the chicken in Korea during 2000 to 2008. Poult Sci 89:236–242 20075274 10.3382/ps.2009-00420

[7] Huang XY, Ansari AR, Huang HB, Zhao X, Li NY, Sun ZJ, Peng KM, Zhong J, Liu HZ (2017) Lipopolysaccharide mediates immuno-pathological alterations in young chicken liver through TLR4 signaling. BMC Immunol 18:12 28241791 10.1186/s12865-017-0199-7 PMC5327529

[8] Brandenburg K, Wiese A (2004) Endotoxins: relationships between structure, function, and activity. Curr Top Med Chem 4:1127–1146 15279605 10.2174/1568026043388213

[9] Senevirathne A, Hewawaduge C, Sivasankar C, Lee JH (2022) Prospective lipid-A altered live attenuated *Salmonella* Gallinarum confers protectivity, DIVA capability, safety and low endotoxicity against fowl typhoid. Vet Microbiol 274:109572 36113357 10.1016/j.vetmic.2022.109572

[10] Breazeale SD, Ribeiro AA, McClerren AL, Raetz CR (2005) A formyltransferase required for polymyxin resistance in *Escherichia coli* and the modification of lipid A with 4-Amino-4-deoxy-l-arabinose. Identification and function oF UDP-4-deoxy-4-formamido-l-arabinose. J Biol Chem 280:14154–14167 15695810 10.1074/jbc.M414265200

[11] Senevirathne A, Hewawaduge C, Lee JH (2022) Assessing an O-antigen deficient, live attenuated *Salmonella* gallinarium strain that is DIVA compatible, environmentally safe, and protects chickens against fowl typhoid. Dev Comp Immunol 133:104433 35568244 10.1016/j.dci.2022.104433

[12] Kirthika P, Jawalagatti V, Senevirathne A, Lee JH (2022) Coordinated interaction between Lon protease and catalase-peroxidase regulates virulence and oxidative stress management during salmonellosis. Gut Microbes 14:2064705 35438052 10.1080/19490976.2022.2064705 PMC9037549

[13] Kirthika P, Senevirathne A, Jawalagatti V, Park S, Lee JH (2020) Deletion of the lon gene augments expression of *Salmonella* Pathogenicity Island (SPI)-1 and metal ion uptake genes leading to the accumulation of bactericidal hydroxyl radicals and host pro-inflammatory cytokine-mediated rapid intracellular clearance. Gut Microbes 11:1695–1712 32567462 10.1080/19490976.2020.1777923 PMC7524146

[14] Matsuda K, Chaudhari AA, Kim SW, Lee KM, Lee JH (2010) Physiology, pathogenicity and immunogenicity of lon and/or cpxR deleted mutants of *Salmonella* Gallinarum as vaccine candidates for fowl typhoid. Vet Res 41:59 20487719 10.1051/vetres/2010031 PMC2887653

[15] Aganja RP, Sivasankar C, Hewawaduge C, Lee JH (2022) Safety assessment of compliant, highly invasive, lipid A-altered, O-antigen-defected *Salmonella* strains as prospective vaccine delivery systems. Vet Res 53:76 36183131 10.1186/s13567-022-01096-z PMC9526937

[16] Ishiguro A, Nishioka M, Morishige A, Kawano R, Kobayashi T, Fujinaga A, Takagi F, Kogo T, Morikawa Y, Okayama N, Mizuno H, Aihara M, Suehiro Y, Yamasaki T (2020) What is the best wavelength for the measurement of hemolysis index? Clin Chim Acta 510:15–20 32621815 10.1016/j.cca.2020.06.046

[17] Guo R, Jiao Y, Li Z, Zhu S, Fei X, Geng S, Pan Z, Chen X, Li Q, Jiao X (2017) Safety, protective immunity, and DIVA capability of a rough mutant *Salmonella* Pullorum vaccine candidate in broilers. Front Microbiol 8:547 28424675 10.3389/fmicb.2017.00547 PMC5380749

[18] Hewawaduge C, Senevirathne A, Sivasankar C, Lee JH (2023) The impact of lipid A modification on biofilm and related pathophysiological phenotypes, endotoxicity, immunogenicity, and protection of *Salmonella* Typhimurium. Vet Microbiol 282:109759 37104940 10.1016/j.vetmic.2023.109759

[19] Bertram EM, Jilbert AR, Kotlarski I (1997) Optimization of an in vitro assay which measures the proliferation of duck T lymphocytes from peripheral blood in response to stimulation with PHA and ConA. Dev Comp Immunol 21:299–310 9258611 10.1016/s0145-305x(97)00005-0

[20] Aganja RP, Sivasankar C, Lee JH (2023) AI-2 quorum sensing controlled delivery of cytolysin-A by tryptophan auxotrophic low-endotoxic *Salmonella* and its anticancer effects in CT26 mice with colon cancer. J Adv Res 61:83–100 37689243 10.1016/j.jare.2023.09.003 PMC11258660

[21] Datsenko KA, Wanner BL (2000) One-step inactivation of chromosomal genes in *Escherichia coli* K-12 using PCR products. Proc Natl Acad Sci U S A 97:6640–6645 10829079 10.1073/pnas.120163297 PMC18686

[22] Ojima S, Okamura M, Osawa N, Tamura A, Yoshioka K, Kashimoto T, Haneda T, Ono HK, Hu DL (2021) Characteristics of systemic infection and host responses in chickens experimentally infected with *Salmonella enterica* serovar Gallinarum Biovar Gallinarum. J Vet Med Sci 83:1147–1154 34039786 10.1292/jvms.21-0227 PMC8349805

[23] Beylefeld A, Abolnik C (2023) *Salmonella* gallinarum strains from outbreaks of fowl typhoid fever in Southern Africa closely related to SG9R vaccines. Front Vet Sci 10:1191497 37476827 10.3389/fvets.2023.1191497 PMC10354334

[24] Van Immerseel F, Studholme DJ, Eeckhaut V, Heyndrickx M, Dewulf J, Dewaele I, Van Hoorebeke S, Haesebrouck F, Van Meirhaeghe H, Ducatelle R, Paszkiewicz K, Titball RW (2013) *Salmonella* Gallinarum field isolates from laying hens are related to the vaccine strain SG9R. Vaccine 31:4940–4945 10.1016/j.vaccine.2013.08.033 23994381

[25] Kwon HJ, Cho SH (2011) Pathogenicity of SG 9R, a rough vaccine strain against fowl typhoid. Vaccine 29:1311–1318 21134445 10.1016/j.vaccine.2010.11.067

[26] Yang KH, Lee MG (2008) Effects of endotoxin derived from *Escherichia coli* lipopolysaccharide on the pharmacokinetics of drugs. Arch Pharm Res 31:1073–1086 18806948 10.1007/s12272-001-1272-8

[27] Kawasaki K (2012) Complexity of lipopolysaccharide modifications in: its effects on endotoxin activity, membrane permeability, and resistance to antimicrobial peptides. Food Res Int 45:493–501

[28] Kawasaki K, Ernst RK, Miller SI (2005) Inhibition of *Salmonella enterica* serovar typhimurium lipopolysaccharide deacylation by aminoarabinose membrane modification. J Bacteriol 187:2448–2457 15774888 10.1128/JB.187.7.2448-2457.2005 PMC1065228

[29] Kawasaki K, Ernst RK, Miller SI (2004) 3-*O*-deacylation of lipid A by PagL, a PhoP/PhoQ-regulated deacylase of *Salmonella* typhimurium, modulates signaling through toll-like receptor 4. J Biol Chem 279:20044–20048 15014080 10.1074/jbc.M401275200

[30] Ernst RK, Guina T, Miller SI (2001) *Salmonella* typhimurium outer membrane remodeling: role in resistance to host innate immunity. Microbes Infect 3:1327–1334 11755422 10.1016/s1286-4579(01)01494-0

[31] Raetz CR, Whitfield C (2002) Lipopolysaccharide endotoxins. Annu Rev Biochem 71:635–700 12045108 10.1146/annurev.biochem.71.110601.135414 PMC2569852

[32] Park BS, Song DH, Kim HM, Choi BS, Lee H, Lee JO (2009) The structural basis of lipopolysaccharide recognition by the TLR4-MD-2 complex. Nature 458:1191–1195 19252480 10.1038/nature07830

[33] Agrawal RK, Singh BR, Babu N, Chandra M (2005) Novel haemolysins of *Salmonella enterica* spp. *Enterica* Serovar Gallinarum. Indian J Exp Biol 43:626–630 16053269

[34] Ferenci T (1999) Growth of bacterial cultures’ 50 years on: towards an uncertainty principle instead of constants in bacterial growth kinetics. Res Microbiol 150:431–438 10540906 10.1016/s0923-2508(99)00114-x

[35] Mukherjee S, Bassler BL (2019) Bacterial quorum sensing in complex and dynamically changing environments. Nat Rev Microbiol 17:371–382 30944413 10.1038/s41579-019-0186-5 PMC6615036

[36] Doublet B, Douard G, Targant H, Meunier D, Madec JY, Cloeckaert A (2008) Antibiotic marker modifications of lambda red and FLP helper plasmids, pKD46 and pCP20, for inactivation of chromosomal genes using PCR products in multidrug-resistant strains. J Microbiol Methods 75:359–361 18619499 10.1016/j.mimet.2008.06.010

