# Supplementary figures and images for: Deletion of pagL and arnT genes involved in LPS structure and charge modulation in the Salmonella genome confer reduced endotoxicity and retained efficient protection against wild-type Salmonella Gallinarum challenge in chicken
Source: Vet Res. 2025 Jan 4;56:2. doi: 10.1186/s13567-024-01413-8 (PMC11699673; doi:10.1186/s13567-024-01413-8)

**Additional file 1.** **Confirmation of deletion of *lon, rfaL, pagL,* and *arnT* genes.**


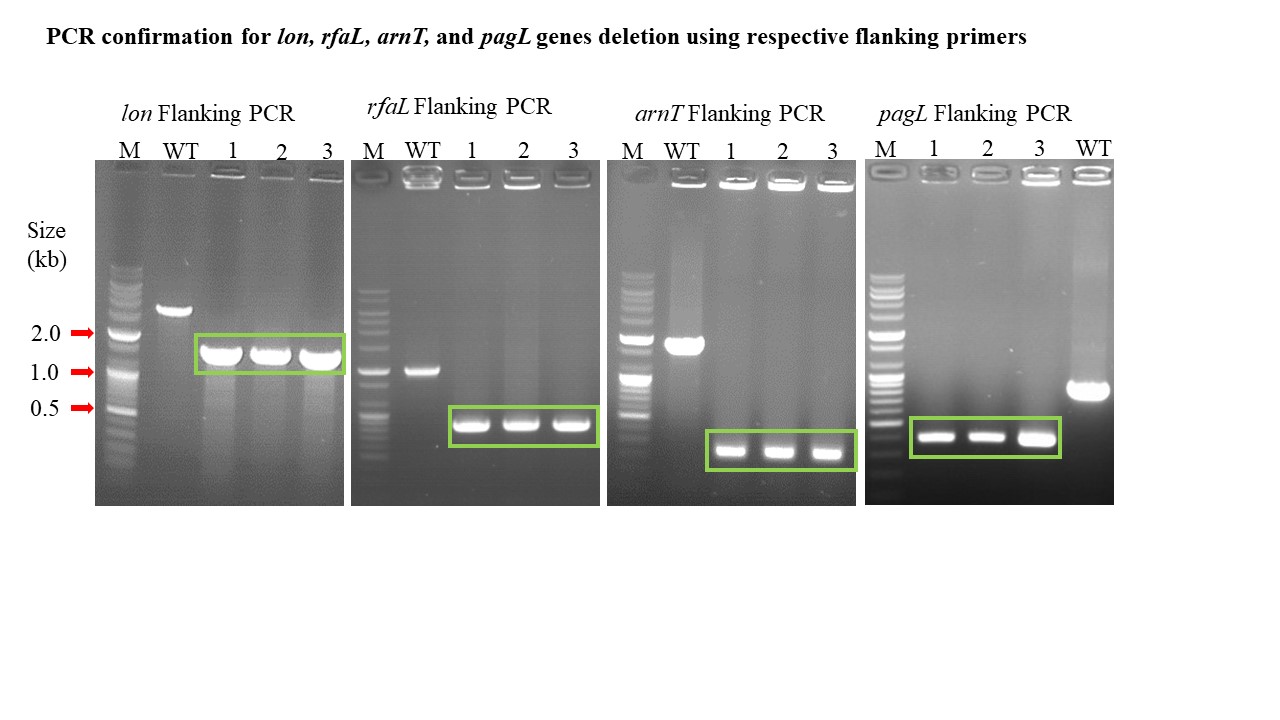

Supplement: Supplementary file 1 — Additional file 1. Confirmation of deletion of lon , rfaL , pagL , and arnT genes. Flanking primers were used to confirm the deletion of respective genes. M = DNA marker, WT = Wild-type, and 1, 2, and 3 = Samples. [file 13567_2024_1413_MOESM1_ESM.docx]
