# Supplementary material for: Deletion of pagL and arnT genes involved in LPS structure and charge modulation in the Salmonella genome confer reduced endotoxicity and retained efficient protection against wild-type Salmonella Gallinarum challenge in chicken
Source: Vet Res. 2025 Jan 4;56:2. doi: 10.1186/s13567-024-01413-8 (PMC11699673; doi:10.1186/s13567-024-01413-8)

**Additional file 2. Gating strategy used for T cell subsets, a representative sample for JOL3016 group.** A. Gating of Total lymphocytes. B. Gating of CD3+ T cells from total lymphocytes. C. Gating of CD3+CD4+ and CD3+CD+ T cells from CD3+ T cells.


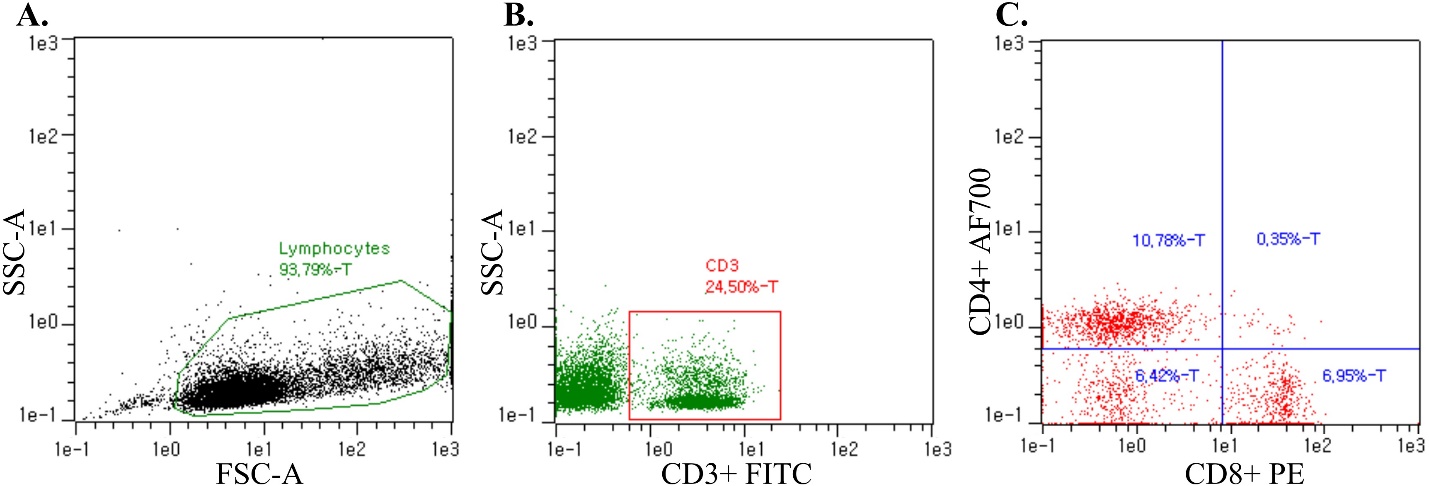

Supplement: Supplementary file 2 — Additional file 2. Gating strategy used for T-cell subsets , a representative sample for the JOL3016 group. (A) Gating of Total lymphocytes. (B) Gating of CD3 + T cells from total lymphocytes. (C) Gating of CD3 + CD4 + and CD3 + CD + T cells from CD3 + T cells. [file 13567_2024_1413_MOESM2_ESM.docx]
