# Supplementary material for: Deletion of pagL and arnT genes involved in LPS structure and charge modulation in the Salmonella genome confer reduced endotoxicity and retained efficient protection against wild-type Salmonella Gallinarum challenge in chicken
Source: Vet Res. 2025 Jan 4;56:2. doi: 10.1186/s13567-024-01413-8 (PMC11699673; doi:10.1186/s13567-024-01413-8)
Supplement: Supplementary file 3 — Additional file 3. Measurement of body temperature (°C) at post-immunisation. [file 13567_2024_1413_MOESM3_ESM.docx]

**Additional file 3.** **Measurement of body temperature (°C) at post-immunization**

| Stage | 1° immunization | | 2° immunization | |  | Challenge | | |
| --- | --- | --- | --- | --- | --- | --- | --- | --- |
| Group/Age | W4 | W5 | W6 | W7 | W8 | W9 | W10 | W11 |
| Naive | 40.7 ± 0.3 | 41.0 ± 0.2 | 40.9 ± 0.3 | 40.8 ± 0.5 | 40.5 ± 0.4 | 40.5 ± 0.4 | 40.2 ± 0.4 | 40.8 ± 0.5 |
| PBS | 40.5 ± 0.4 | 40.9 ± 0.4 | 40.8 ± 0.3 | 41.1 ±0.2 | 40.6 ±0.3 | **42.1 ±0.2** | **42.3 ± 0.3** |  |
| SG9R | 41.3 ± 0.3 | 41.2 ± 0.4 | 41.4 ± 0.4 | 41.5 ± 0.3 | 41.3 ± 0.2 | 41.5 ± 0.2 | 41.1 ± 0.4 | 40.9 ± 0.4 |
| JOL3015 | 41.0 ± 0.3 | 40.8 ± 0.4 | 41.2 ± 0.2 | 40.9 ± 0.2 | 40.8 ± 0.2 | 41.3 ± 0.5 | 41.1 ± 0.3 | 41.0 ± 0.2 |
| JOL3016 | 41.2 ± 0.2 | 41.0 ± 0.3 | 41.5 ± 0.3 | 40.8 ± 0.4 | 40.6 ± 0.3 | 41.1 ± 0.2 | 40.9 ± 0.3 | 40.9 ± 0.2 |

Note: W4; 4 Weeks Old, W5; 5 Weeks Old, W6; 6 Weeks Old, W7; 7 Weeks Old, W8, 8 Weeks Old, W9; 9 Weeks Old, W10; 10 Weeks Old, and W11; 11 Weeks Old
